# Generalized temporal transfer matrix method: a systematic approach to solving electromagnetic wave scattering in temporally stratified structures

**DOI:** 10.1515/nanoph-2021-0715

**Published:** 2022-03-07

**Authors:** Jingwei Xu, Wending Mai, Douglas H. Werner

**Affiliations:** Department of Electrical Engineering, The Pennsylvania State University, University Park, PA 16802, USA

**Keywords:** anisotropy; anti-reflection coating; multi-layer medium; polarization conversion, temporal modulation, transfer matrix method

## Abstract

Opening a new door to tailoring electromagnetic (EM) waves, temporal boundaries have attracted the attention of researchers in recent years, which have led to many intriguing applications. However, the current theoretical approaches are far from enough to handle the complicated temporal systems. In this paper, we develop universal matrix formalism, paired with a unique coordinate transformation technique. The approach can effectively deal with temporally stratified structures with complicated material anisotropy and arbitrary incidence angles. This formulation is applied to various practical systems, enabling the solution of these temporal boundary related problems in a simple and elegant fashion, and also facilitating a deep insight into the fundamental physics.

## 1 Introduction

Time-varying metamaterials and metasurfaces facilitate a new degree of freedom for controlling electromagnetic (EM) waves. In recent years, significant efforts have been devoted to this topic, enabling some novel phenomena such as non-reciprocity, frequency conversion [1], [2], [3], [4], [5], dispersion engineering [6], asymmetric propagation [7], bandwidth extension [8], harmonic information transition [9], time-varying optical vortices [10], and spectrum spreading [11]. These characteristics typically can’t be achieved with conventional metamaterials and metasurfaces that are time-invariant and designed to operate in the frequency domain. Active components are usually required in order to bestow metasurfaces with the desired time-modulation, such as lumped elements [1], real-time interference patterns [2], and optical pumping [3].

**Figure 1:**
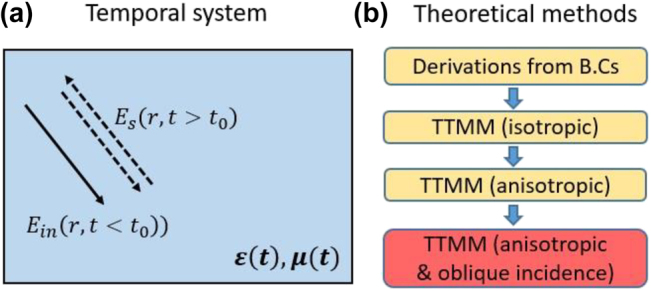
(a) Schematics of temporal boundary value problems (TBVPs). (b) Flowchart showing the current theoretical approaches for the system described in (a) where the contribution of this paper is highlighted in red.

Despite the significant achievements made by researchers, the time modulation is usually confined to a small volume (i.e., within metasurface unit cells), and consequently the desired phenomena, such as frequency conversion or harmonic transition, are still characterized in the time-invariant regime. One may be naturally curious, however, regarding what would happen if the time modulation were to occur over a much larger region. In [12], the authors proposed the revolutionary concept of a temporal boundary. Their work describes an EM wave propagating in an infinite homogeneous medium (see Figure 1a), whose material parameters $\epsilon_{r}$ or $\mu_{r}$ experience a sudden change at an instant of time $t=t_{0}$ . Using boundary conditions, one can prove that the wave will split in two, where each wave travels in an opposite direction at $t=t_{0}$ . This phenomenon is actually a temporal dual of the well-known reflection and transmission phenomenon that occurs at a spatial interface between two media.

Having been explored theoretically and numerically, this concept soon mushroomed into a rich topic of research over the past several years. Many concepts have emerged that rely on tailoring waves at spatial boundaries and transforming them to the temporal domain. They include effective medium theory [13, 14], anti-reflection coatings [15], Fabry–Perot cavities [16, 17], prisms [18], waveguides [19, 20], photonic crystals [21], [22], [23], polarization conversion [24], total internal reflection [25], the Brewster angle [26], parity-time (PT) symmetry [27], and impedance transformers [28]. Moreover, by utilizing temporal boundaries, some ideas unique to temporal systems have also been explored. For example, in [29], the authors were able to achieve a real-time redirection of energy propagation. The notion of EM cloaks was generalized in [30], so that an ‘event’, rather than an object, could be concealed. In [31], it was found that there is an exponential increase of intensity when a wave travels through a temporally disordered structure. The energy conservation issue associated with a pulse travelling through a temporal boundary is investigated in [32]. Finally, in [33, 34], the authors studied the properties of temporal discontinuities in dispersive media. These concepts and associated designs result from solving the appropriate Temporal Boundary Value Problem (TBVP): a terminology which we adopt in our later discussions.

While these applications bring new opportunities in optics and electromagnetics, many of them involve complex temporal systems, including multiple (or even infinite) temporal boundaries [13], [14], [15], [16, 21], [22], [23], [24, 28] and anisotropic materials [18, 24, 26, 29]. Thus, the formulation introduced in [12], which is targeted to an EM wave’s reflection and transmission near a single temporal boundary, while revolutionary, is quite limited in terms of its applicability to solving more general TBVPs. Much of the literature [13, 15, 16, 18, 26, 29] relies on direct derivations from the boundary conditions of Maxwell’s equations in order to calculate the desired quantities, such as *S* parameters. In fact, all of the derivations are based on the same principle; consequently, they are inevitably repetitive, and sometimes lengthy. In some cases considered in the literature [22, 31], the temporal systems are too complex to have a closed form solution. Therefore, the important question arises; can an overarching theoretical approach be developed to handle all the TBVPs?

Indeed, there are some theoretical works that have attempted to address EM wave propagation inside materials with arbitrary $\epsilon_{r}\left(t\right)$ or $\mu_{r}\left(t\right)$ profiles using WKB approximations [35, 36]. Though powerful, this approach is not very suitable for TBVPs, where the material properties only undergo abrupt changes. The dual-nature of spatial and temporal boundaries suggests that the transfer matrix method (TMM) [37] would be an ideal candidate for modeling such problems, since it represents a powerful tool for analyzing the scattering of EM waves in multilayer structures. In [38], the authors extended the idea of the TMM to the time domain, and developed a formalism, which could be used to calculate the responses of waves to ‘a multilayer temporal structure’. This method, though effective in some cases, is intrinsically limited, because it is only applicable to isotropic materials and normally incident waves. Some other works have also employed preliminary forms of Temporal TMM (i.e., TTMM) to solve specific problems, but were not extended to tackle other similar systems [17, 21, 27, 39]. In our previous work [24], we were inspired by the research reported in [40], and developed a more complex formalism that could handle anisotropic material systems. However, our previous approach did not incorporate the important general case where the incident wave is oblique, thus it is incapable of being used to solve the systems as described in [18, 26, 29, 41].

In light of this shortcoming, in this paper, we introduce a theoretical framework that can handle EM wave interactions with homogenous time-variant materials, for arbitrary incident angles, and material anisotropy. First, we demonstrate that the concept of ‘oblique incidence’ needs to be clarified and re-defined, which poses a challenge in solving problems with temporal boundaries. Then, we adopt a coordinate transformation strategy in order to address this issue, and develop a method that we call generalized TTMM (GTTMM). Using this method, we successfully analyze EM wave responses in several practical temporal systems. Moreover, numerical simulations are also performed to validate the analytical results.

## 2 Challenges

**Figure 2:**
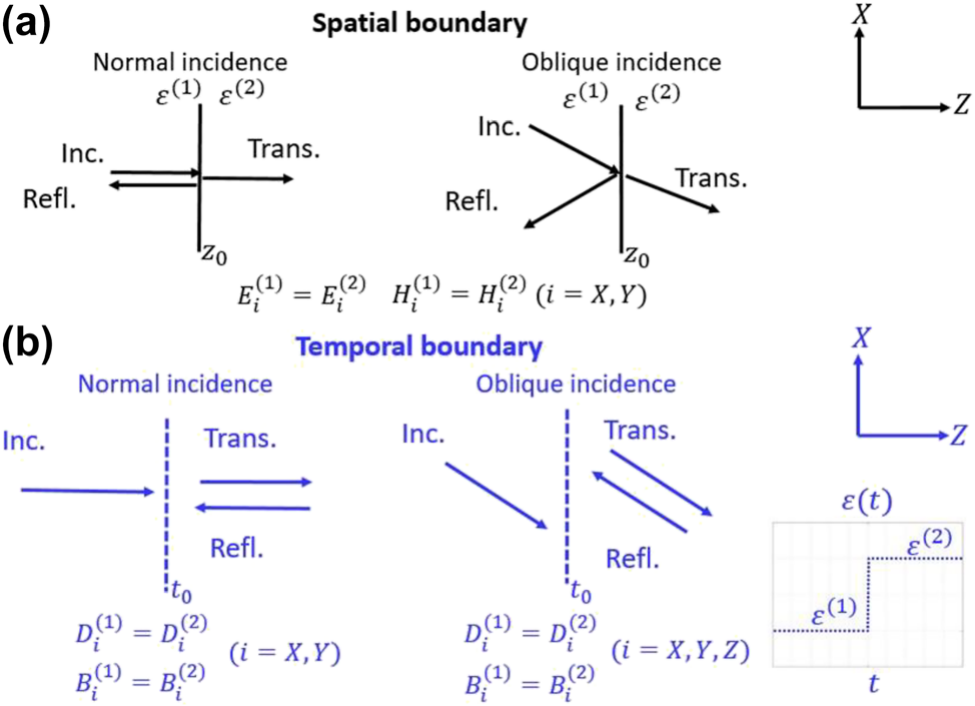
Schematics showing the difference between (a) spatial and (b) temporal boundaries, in terms of the associated boundary conditions.

First, we revisit the conventional 4×4 TMM formalism used in the spatial scenario [40]. It is instructive to ask the question: What is the origin of the number ‘4’? In order to answer this, let us consider a spatial domain that is composed of two media for simplicity, which have an interface at $Z=Z_{0}$ (see Figure 2a). Moreover, we assume that the anisotropy of these media has principal axes XYZ (i.e., in XYZ coordinates, the permittivity and permeability tensors are diagonal matrices). According to Maxwell’s equations, the tangential components of the E and H fields are continuous at $Z_{0}$ [7] and, consequently, there are four independent equations to solve:

(1) $$E_{X}^{\left(1\right)}=E_{X}^{\left(2\right)},E_{Y}^{\left(1\right)}=E_{Y}^{\left(2\right)},H_{X}^{\left(1\right)}=H_{X}^{\left(2\right)},H_{Y}^{\left(1\right)}=H_{Y}^{\left(2\right)}$$

Hence, in this formalism, the transfer matrix needs to have a dimension of four in order to fully describe the properties of the EM system. Equation (1) is valid no matter whether the wave is obliquely or normally incident, because the normal to the interface is always along the Z direction.

Now let us consider a temporal scenario such that the wave is travelling in a homogeneous material, which changes suddenly from medium 1 to 2 (see Figure 2b). In this case, however, the boundary conditions of Maxwell’s equations require that all three components of the D and B fields must be continuous:

(2) $$D_{i}^{\left(1\right)}=D_{i}^{\left(2\right)},B_{i}^{\left(1\right)}=B_{i}^{\left(2\right)}\left(i=X,Y,Z\right)$$

Here, there are six independent equations which must be solved simultaneously. Obviously, the classical 4×4 TMM formalism is not applicable in this case. The fundamental reason for this lies in the difference between Eq. (1) and (2). That is, the existence of the temporal boundary does not define a special direction in space. In our previous work [24], we assumed that the wave travels in the +Z direction. Under this special circumstance, Eq. (2) reduces to:

(3) $$D_{X}^{\left(1\right)}=D_{X}^{\left(2\right)},D_{Y}^{\left(1\right)}=D_{Y}^{\left(2\right)},B_{X}^{\left(1\right)}=B_{X}^{\left(2\right)},B_{Y}^{\left(1\right)}=B_{Y}^{\left(2\right)}$$

With this assumption, the formalism presented in [40] could be easily adapted to produce a temporal counterpart, because the number of independent equations is reduced to 4. However, what happens if the wave does not propagate in +Z direction?

**Figure 3:**
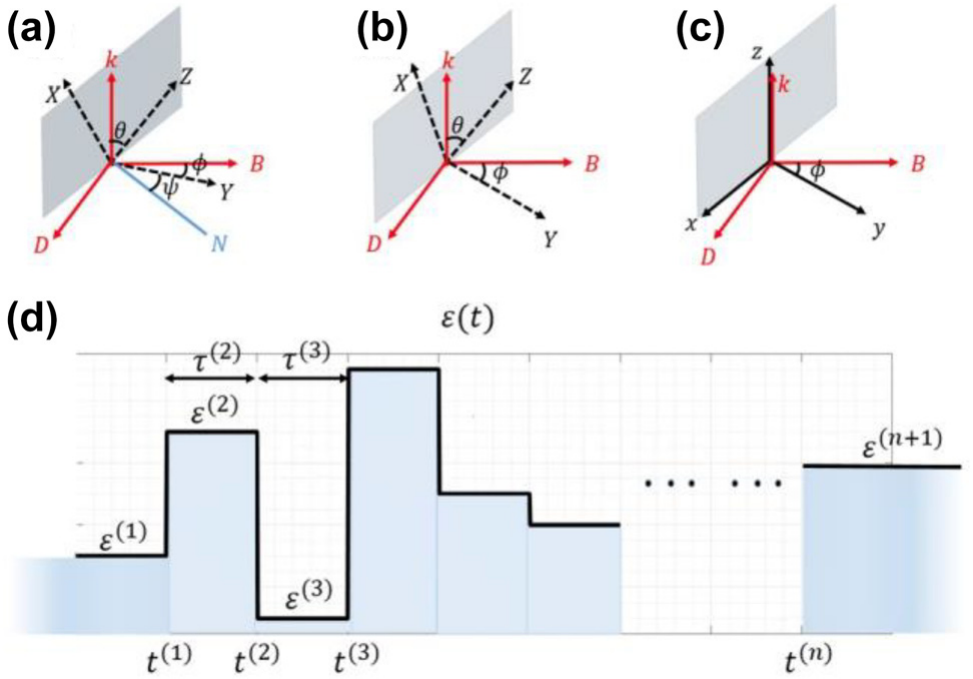
(a)–(c) Schematics illustrating the coordinate transformation. (a) The Euler angle representation of S1 and S2. N is defined as the normal direction of the k−Z plane. (b) The simplification of (a) with ψ=0. (c) S1 after rotation (S3). (d) The temporal profile of the material system.

In order to address this dilemma, we resort to a ‘coordinate transformation technique’. Specifically, the anisotropy of the material and the incident wave define two coordinate systems: X−Y−Z and k−D−B , which we refer to as S1 and S2. From the theory of rigid bodies [42], one requires three independent parameters θ , ϕ , and ψ (i.e., Euler angles) to fully describe the relationship between the two sets of coordinates, as schematically depicted in Figure 3a. Importantly, without a spatial interface, there is no such thing as ‘incident angles’ in the traditional sense for such temporal systems. In this paper, we define θ as the incidence angle, thus, normal and oblique incidence correspond to θ=0 and θ≠0 , respectively. One can also find that this definition only makes sense if the material is anisotropic. This represents a significant difference between temporal and spatial boundaries.

At this point, for simplicity, a special case: ψ=0 , is assumed for all the following discussions (see Figure 3b). We note that if ψ≠0 , the dispersion relation would be extremely complicated. Next, we rotate S1 along the Y axis so that Z and k will overlap, and *X* and *Y* would lie in the D−B plane. This new coordinate system is called ‘ x−y−z ’, or S3 (see Figure 3c). Observing from S3, D and B would only have x and y components. If we further define the fields and associated *S* parameters in terms of D rather than E (this will be elaborated upon later), then our previously proposed method [24] can be easily adapted and generalized to the oblique incidence case.

## 3 Theoretical formulation

### 3.1 TTMM formalism

Before establishing the TTMM formalism, let us define the temporal system (Figure 3d). It consists of an unbounded homogenous medium, whose permittivity or permeability undergoes abrupt changes n times at $t^{\left(i\right)}\left(i=1,2,3\ldots n\right)$ . It can be regarded as a ‘temporal stratified structure’ consisting of n−1 temporal layers, whose durations are $\tau^{\left(i\right)}=t^{\left(i\right)}-t^{\left(i-1\right)}\left(i=2,3,4\ldots n\right)$ . Moreover, an EM wave is incident before $t^{\left(1\right)}$ .

Several assumptions are made about the system: (a) The anisotropy of each temporal layer shares the same principal axes ( XYZ ), and (b) in the first temporal layer (i.e., when the wave is incident), the material is isotropic, which guarantees that k, D , and B are perpendicular to each other. These two assumptions are the foundations of the coordinate transformation. In S1, the material property in the i -th layer is expressed as

$${\epsilon^{\prime }}^{\left(i\right)}=\text{diag}\left(\epsilon_{X}^{\left(i\right)},\epsilon_{Y}^{\left(i\right)},\epsilon_{Z}^{\left(i\right)}\right)\;$$

(4.1) $$\mu^{\prime \left(i\right)}=\text{diag}\left(\mu_{X}^{\left(i\right)},\mu_{Y}^{\left(i\right)},\mu_{Z}^{\left(i\right)}\right)$$

where $\epsilon^{\prime \left(i\right)}$ and $\mu^{\prime \left(i\right)}$ denote relative permittivity and permeability (here, and in all of the following discussions, the subscript ‘*r*’ is dropped, for simplicity). Notice that the explicit expressions for ϵ and μ depend on the choice of coordinate system. Therefore, in S3, we have

(4.2) $$\epsilon^{\left(i\right)}=A^{-1}\epsilon^{\prime \left(i\right)}A\;\mu^{\left(i\right)}=A^{-1}\mu^{\prime \left(i\right)}A$$

where A is the rotation matrix between S1 and S3:

(4.3) $$\left(\begin{array}{lll} \text{cos}\left(\theta \right) & 0 & -\text{sin}\left(\theta \right)\\ 0 & 1 & 0\\ \text{sin}\left(\theta \right) & 0 & \text{cos}\left(\theta \right) \end{array}\right)$$

Notice that $\epsilon^{\left(i\right)}$ and $\mu^{\left(i\right)}$ have off-diagonal terms. For the remainder of this section, all the physical quantities and expressions derived are considered in the framework of the S3 coordinate system, unless specified otherwise. Based on Maxwell’s equations, the D field should satisfy the relation:

(5) $$k\times \left[\mu_{0}\mu^{-1}\left(k\times \left(\epsilon_{0}\epsilon^{-1}D\right)\right)\right]+\frac{\omega^{2}}{c^{2}}D=0$$

where $k=\left(k_{\text{x}},k_{\text{y}}\right)$ is the wave vector (a known, unchanged value in homogenous media), and ω is the radian frequency.

Similar to the discussions in [24, 40], one can express the D and B field in the i -th layer as linear combinations of the four modes:

(6.1) $$\left(\begin{array}{l} D^{\left(i\right)}\\ B^{\left(i\right)} \end{array}\right)=\overset{4}{\underset{\sigma =1}{\sum }}A_{\sigma }^{\left(i\right)}\left(\begin{array}{l} d_{\sigma }^{\left(i\right)}\\ b_{\sigma }^{\left(i\right)} \end{array}\right){\text{e}}^{-i\left[k\cdot r-\omega_{\sigma }^{\left(i\right)}\left(t-t^{\left(i\right)}\right)\right]}$$

where { $A_{1}^{\left(i\right)},A_{2}^{\left(i\right)},A_{3}^{\left(i\right)},A_{4}^{\left(n\right)}$ } are a set of expansion coefficients of the modes. The terms $d_{\sigma }^{\left(i\right)}$ and $b_{\sigma }^{\left(i\right)}$ denote the unit field vectors of ***D*** and ***B*** inside the i -th layer. According to Maxwell’s equations, the following relation is satisfied:

(6.2) $$b_{\sigma }^{\left(i\right)}=\frac{1}{\omega_{\sigma }^{\left(i\right)}}\left(k\times {\left(\epsilon_{0}\epsilon^{\left(i\right)}\right)}^{-1}d_{\sigma }^{\left(i\right)}\right)$$

By applying the boundary conditions at the i -th temporal boundary:

(7) $$\left(\begin{array}{l} D^{\left(i\right)}\\ B^{\left(i\right)} \end{array}\right)=\left(\begin{array}{l} D^{\left(i+1\right)}\\ B^{\left(i+1\right)} \end{array}\right)$$

we have

(8) $$D_{i+1}\left(\begin{array}{l} A_{1}^{\left(i+1\right)}\\ A_{2}^{\left(i+1\right)}\\ A_{3}^{\left(i+1\right)}\\ A_{4}^{\left(i+1\right)} \end{array}\right)=D_{i}P_{i}\left(\begin{array}{l} A_{1}^{\left(i\right)}\\ A_{2}^{\left(i\right)}\\ A_{3}^{\left(i\right)}\\ A_{4}^{\left(i\right)} \end{array}\right)$$

where $P_{n}$ is the temporal propagation matrix defined as (Eq. S5 in [24]):

$$P_{i}=\text{diag}\left(P_{i,1},P_{i,2},P_{i,3},P_{i,4}\right)$$

(9) $$P_{i,\sigma }=\exp\left(i\omega_{\sigma }^{\left(i\right)}\tau^{\left(i\right)}\right),\sigma =1,2,3,4$$

and $D_{i}$ is defined as (similar to Eq. S4 in [24] and Eq. (14) in [40]):

(10) $$D_{i}=\left(\begin{array}{llll} d_{1}^{\left(i\right)}\cdot y & d_{2}^{\left(i\right)}\cdot y & d_{3}^{\left(i\right)}\cdot y & d_{4}^{\left(i\right)}\cdot y\\ b_{1}^{\left(i\right)}\cdot x & b_{2}^{\left(i\right)}\cdot x & b_{3}^{\left(i\right)}\cdot x & b_{4}^{\left(i\right)}\cdot x\\ b_{1}^{\left(i\right)}\cdot y & b_{2}^{\left(i\right)}\cdot y & b_{3}^{\left(i\right)}\cdot y & b_{4}^{\left(i\right)}\cdot y\\ d_{1}^{\left(i\right)}\cdot x & d_{2}^{\left(i\right)}\cdot x & d_{3}^{\left(i\right)}\cdot x & d_{4}^{\left(i\right)}\cdot x \end{array}\right)$$

Importantly, at this point in the development, it is not clear what role the incidence angle would play in a generalized formulation. In the following section, however, we will see that when the wave is obliquely incident (i.e., S1 and S3 do not overlap), the relation between k and $\omega_{\sigma }^{\left(i\right)}$ will be different. Therefore, the explicit form of the dispersion relation (Eq. (5)) and the expression for the matrix $D_{n}$ (Eq. (10)) will also change accordingly.

### 3.2 Explicit form of the transfer matrices

First, let us investigate the dispersion relation, as determined by Eq. (5). For each temporal layer, we replace ϵ and μ with $\epsilon^{\left(i\right)}$ and $\mu^{\left(i\right)}$ in Eq. (5), then replace D with $D^{\left(i\right)}$ as defined in Eq. (6.1). It can be shown that there are four non-trivial solutions of the dispersion relation: $k∼\omega_{\sigma }^{\left(i\right)}\;\left(\sigma =1,2,3,4\right)$ . After carrying out the appropriate mathematical analysis (see Supplemental Document 1A), we arrive at

(11.1) $$\omega_{1}^{\left(i\right)}=-\omega_{2}^{\left(i\right)}=ck\sqrt{\frac{c_{1}^{2}\mu_{Z}^{\left(i\right)}+s_{1}^{2}\mu_{X}^{\left(i\right)}}{\epsilon_{Y}^{\left(i\right)}\mu_{X}^{\left(i\right)}\mu_{Z}^{\left(i\right)}}}=\frac{ck}{n_{y}^{\prime \left(i\right)}}$$

(11.2) $$\omega_{3}^{\left(i\right)}=-\omega_{4}^{\left(i\right)}=ck\sqrt{\frac{c_{1}^{2}\epsilon_{Z}^{\left(i\right)}+s_{1}^{2}\epsilon_{X}^{\left(i\right)}}{\epsilon_{X}^{\left(i\right)}\epsilon_{Z}^{\left(i\right)}\mu_{Y}^{\left(i\right)}}}=\frac{ck}{n_{x}^{\prime \left(i\right)}}$$

where $c_{1}=\cos\left(\theta \right)$ and $s_{1}=\text{sin}\left(\theta \right)$ . Also, we introduce the following definitions:

(11.3) $$n_{x}^{\prime \left(i\right)}=\sqrt{\frac{\epsilon_{X}^{\left(i\right)}\epsilon_{Z}^{\left(i\right)}\mu_{Y}^{\left(i\right)}}{c_{1}^{2}\epsilon_{Z}^{\left(i\right)}+s_{1}^{2}\epsilon_{X}^{\left(i\right)}}},n_{y}^{\prime \left(i\right)}=\sqrt{\frac{\epsilon_{Y}^{\left(i\right)}\mu_{X}^{\left(i\right)}\mu_{Z}^{\left(i\right)}}{c_{1}^{2}\mu_{Z}^{\left(i\right)}+s_{1}^{2}\mu_{X}^{\left(i\right)}}}$$

These quantities can be viewed as the equivalent refractive indices seen by *x*- and *y*-polarized waves inside the i -th temporal layer.

It then follows that the corresponding field vectors of D are

(11.4) $$d_{1}^{\left(i\right)}=d_{2}^{\left(i\right)}=\left(0,1,0\right),d_{3}^{\left(i\right)}=d_{4}^{\left(i\right)}=\left(1,0,0\right)$$

Clearly, these four solutions correspond to the two orthogonal modes (i.e., *y*- and *x*-polarized waves) propagating in opposite directions. Next, we use Eqs. (6.2), (10) and (11.4) to obtain:

(12.1) $$D_{i}=\frac{1}{2}\left(\begin{array}{llll} 1 & 1 & 0 & 0\\ \frac{\omega_{1}^{\left(i\right)}\mu_{X}^{\left(i\right)}}{k} & \frac{\omega_{2}^{\left(i\right)}\mu_{X}^{\left(i\right)}}{k} & 0 & 0\\ 0 & 0 & \frac{\omega_{3}^{\left(i\right)}\mu_{Y}^{\left(i\right)}}{k} & \frac{\omega_{4}^{\left(i\right)}\mu_{Y}^{\left(i\right)}}{k}\\ 0 & 0 & 1 & 1 \end{array}\right)$$

which can be rewritten as:

(12.2) $$D_{i}=\frac{1}{2}\left(\begin{array}{llll} 1 & 1 & 0 & 0\\ -Z_{0}Z_{y}^{\prime \left(i\right)} & Z_{0}Z_{y}^{\prime \left(i\right)} & 0 & 0\\ 0 & 0 & Z_{0}Z_{x}^{\prime \left(i\right)} & -Z_{0}Z_{x}^{\prime \left(i\right)}\\ 0 & 0 & 1 & 1 \end{array}\right)$$

At this point we define the following identities:

$$Z_{x}^{\prime \left(i\right)}=\sqrt{\mu_{Y}^{\left(i\right)}\left(\frac{c_{1}^{2}}{\epsilon_{X}^{\left(i\right)}}+\frac{s_{1}^{2}}{\epsilon_{Z}^{\left(i\right)}}\right)}$$

(12.3) $$Z_{y}^{\prime \left(i\right)}=\sqrt{c_{1}^{2}\frac{\mu_{X}^{\left(i\right)}}{\epsilon_{Y}^{\left(i\right)}}+s_{1}^{2}\frac{{\left(\mu_{X}^{\left(i\right)}\right)}^{2}}{\epsilon_{Y}^{\left(i\right)}\mu_{z}^{\left(i\right)}}}$$

which can be viewed as the equivalent impedances seen by the *x*- and *y*-polarized waves inside the i -th temporal layer (normalized by the free space impedance $Z_{0}$ ). It is obvious that, if θ=0 (i.e., normal incidence), then Eq. (12.3) will reduce to the definition of impedance in the normal sense, which is the same as in Eq. (S13) of [24] if i=2 . It is worth mentioning that Eq. (12.1) and (12.2) hold only if the material is anisotropic. If the material is isotropic, the D matrix can take different forms depending on the angle ϕ . This is elaborated on in the Supplemental Document 1B.

With the explicit form of matrices D and P given, one can calculate the total transfer matrix of the temporal structure illustrated in Figure 3d:

(13) $$Q=\left(\overset{2}{\underset{i=n}{\prod }}D_{i+1}^{-1}D_{i}P_{i}\right)D_{2}^{-1}D_{1}=\left(\overset{2}{\underset{i=n}{\prod }}M_{i+1,i}P_{i}\right)M_{21}$$

From the discussion above, we know that the Q matrix is solely determined by ϵ, μ, and the durations of each temporal layer, which is referred to as the (temporal) profile of this temporal structure.

**Figure 4:**
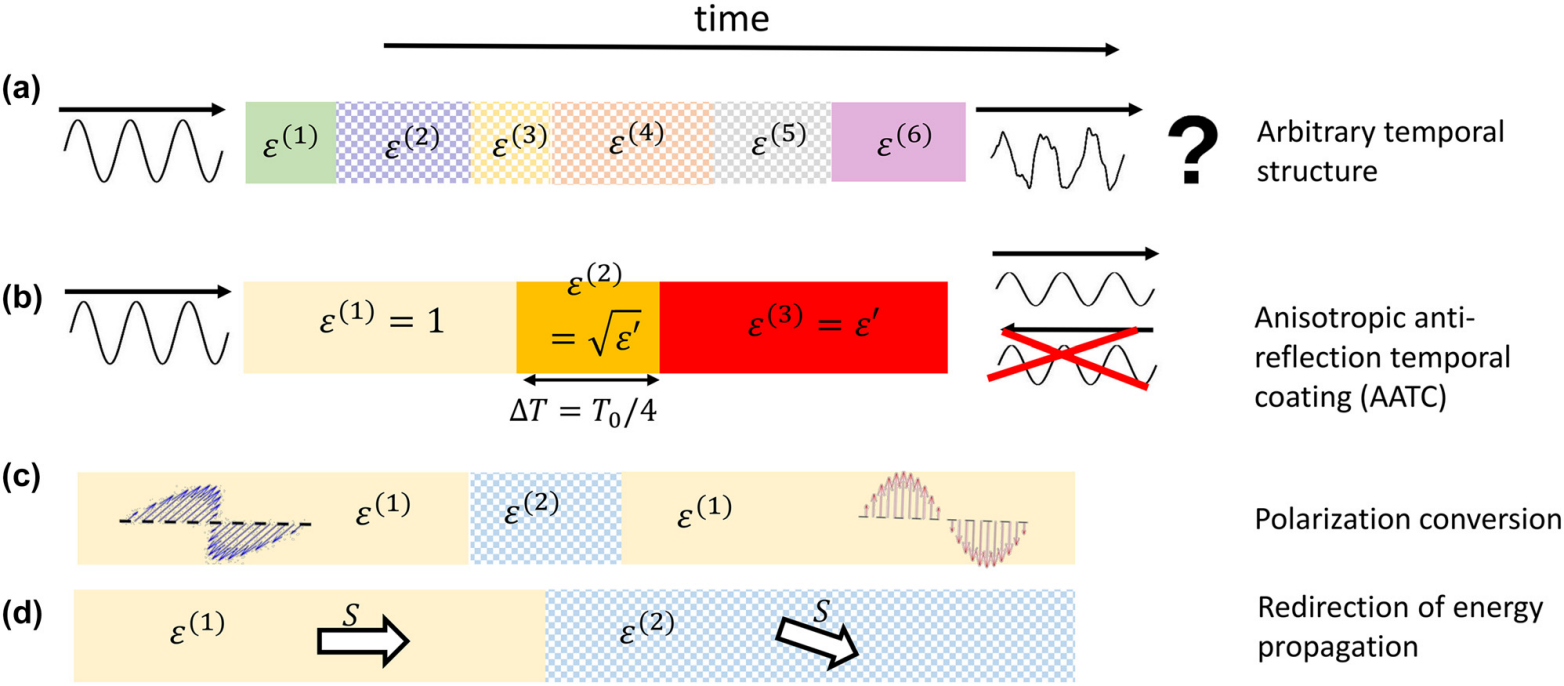
Schematics of several temporal systems. The different temporal regions are represented by colored boxes. The temporal profile in the case of (a) an arbitrary temporal structure, (b) an anisotropic antireflection temporal coating, (c) polarization conversion, and (d) redirection of energy propagation. Notice that anisotropic temporal regions are denoted by checkerboard patterns.

At this point we have completed our introduction to the GTTMM. In the next two sections, we will apply this tool to four different temporal systems, which are schematically represented in Figure 4. All of these systems can be regarded as temporal multilayer structures, as have been described in Figure 3d. Moreover, the desired physical quantities, for example, the permittivity tensor of the anisotropic antireflection temporal coating or the transmission coefficients in the case of polarization conversion, could be calculated directly from the Q matrix. This procedure will be elaborated on in the following sections.

## 4 Application: anisotropic systems

In this section, we will consider some practical TBVPs to demonstrate the application and efficacy of the GTTMM. These TBVPs are relatively complicated, involving multilayer anisotropic temporal structures, which are later simply referred to as ‘structure(s)’. First, in Section A, we introduce a 6-layer structure with a random profile and use it to demonstrate the robustness of our method. Next, in Section 4B, we generalize the idea of an antireflection temporal coating (ATC) [15] to the anisotropic case. In [24], we found that the polarization state of a wave would experience a temporal change if an ‘anisotropic temporal slab’ is ‘inserted’ into an isotropic background medium. Researchers in [25] temporally switched the material permittivity from isotropic to anisotropic, and found that the Poynting vector (i.e., energy flux) of the wave will change in time. Referring to the coordinates illustrated in Figure 3b, the case where θ=0 and ϕ≠0 , was studied in [22], while in [29] the discussion was limited to θ≠0 and ϕ=0 . Here, we extend the work reported in both papers to a more general scenario: θ≠0 and ϕ≠0 , in Section 4C and D, respectively.

For all four examples studied here, we present comparisons of the results obtained from the GTTMM calculations and FDTD simulations. Apart from these examples, we also show that the GTTMM formulation can be reduced to the isotropic scenario as a special case. For verification, we re-derived the results presented in two previously published papers using the GTTMM (see Supplemental Document 2).

**Figure 5:**
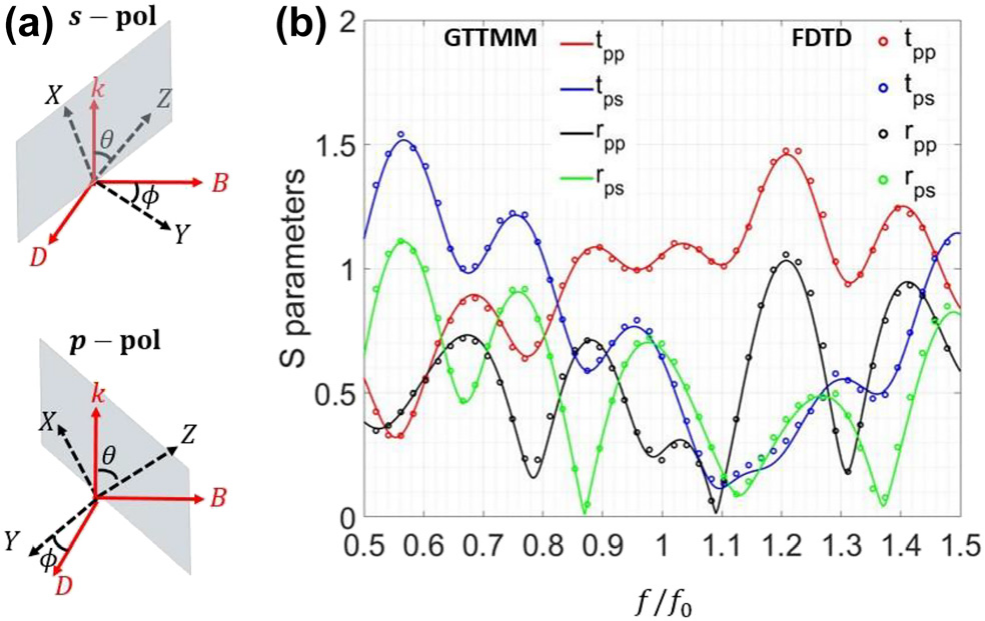
An arbitrary temporal structure: (a) the coordinate representation of two polarization states in the S2 system, where the grey parallelograms denote the XZ plane. (b) *S* parameters, where the GTTMM and FDTD results are represented by solid lines and dots, respectively. $f_{0}$ is the frequency of the incident wave.

### 4.1 Arbitrary multi-layer temporal structure

First, in order to demonstrate the validity of our GTTMM algorithm, we consider a 6-layer structure, which has a random profile. The information on the composition of this structure is displayed in Table 1. Notice that the first and last layers are isotropic, and they are semi-infinite in time. Now, let us study the interactions between the electromagnetic waves and this structure. As mentioned before, two independent parameters, θ and ϕ , are required to determine the orientation of the incident wave. Then we define two orthogonal polarization modes as s and p , as depicted in Figure 5a. The orientation of the latter polarization state can be rotated counter-clockwise from the former one by 90° along k . To this end, any polarization state of the incident, reflected, and transmitted waves can be written as a linear combination of the two modes. Hence, one can naturally define the *S* parameters of a given temporal structure as $t_{ij}$ and $r_{ij}\;\left(i,j=s,p\right)$ . Please refer to Supplemental Document 3 for the detailed calculation methodology for the *S* parameters.

**Table 1:** Profile of the arbitrary 6-layer temporal structure. The first and the last (i.e., the 6th) layers are isotropic materials. The duration of each layer is normalized to $T_{0}=1/f_{0}$ , which is the inverse of the frequency of the incident wave.

Layer	1	2	3	4	5	6
$\epsilon_{xx}$	1	4	8	12	2	1
$\epsilon_{yy}$		13	9	5	6	
$\epsilon_{zz}$		2	7	3	10	
$\Delta t/T_{0}$	NA	1.35	2.22	1.27	1.74	NA

Figure 5 shows the spectral response of a p -polarized wave ‘propagating’ through this temporal structure. The dots and solid lines represent the results obtained by the FDTD simulations and the GTTMM theory, respectively. It can be clearly seen that, for all four S parameters (i.e., $t_{pp}$ , $t_{ps}$ , $r_{pp}$ , $r_{ps}$ ), the numerical and theoretical results match very well over a wide frequency range. Considering the random nature of the temporal structure, this analysis confirms the validity and robustness of the GTTMM. In fact, if the material becomes isotropic, then this case would reduce to Example 1 in [38].

### 4.2 Polarization conversion

**Figure 6:**
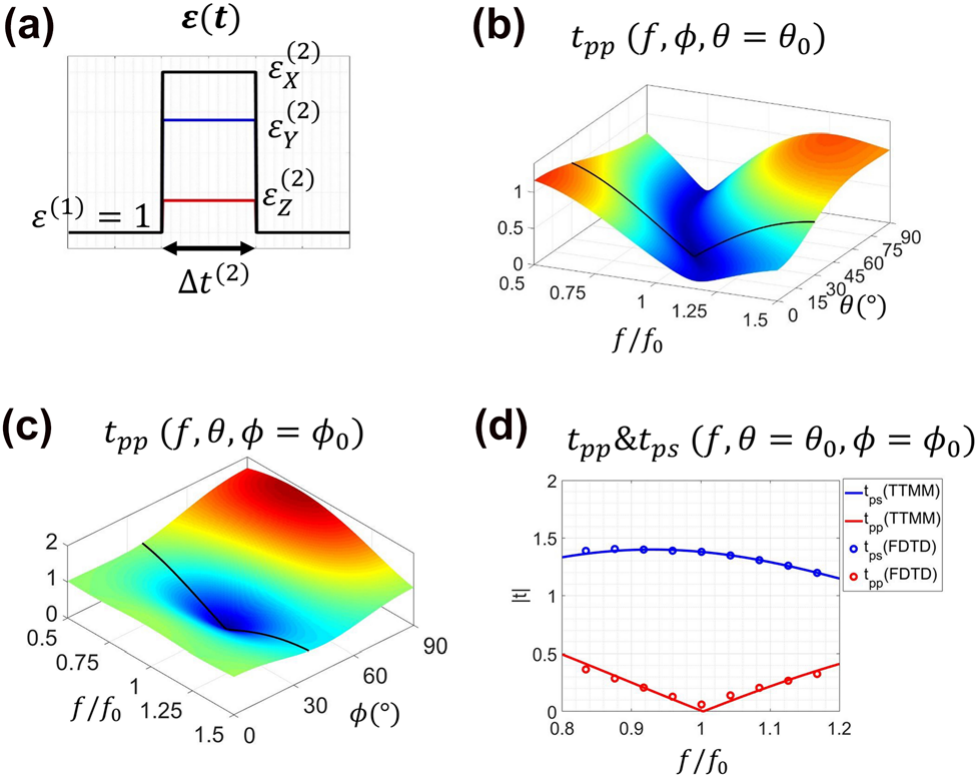
Illustrations of the polarization conversion effect. (a) Schematic of the temporal profile of the system (b)–(d) the transmission spectra.

Polarization conversion is an important property with many practical applications in optics and electromagnetics. Traditionally, one could convert polarization of an EM wave by utilizing the interface between an isotropic and an anisotropic material [43]. In our previous work [24], we extended this idea to the temporal domain, and achieved complete polarization conversions in real time, as schematically demonstrated in Figure 4c. In that work, however, we only considered the normal incidence case ( θ=0 ). What if the wave is obliquely incident ( θ≠0 )? Specifically, we study the temporal system which has a material profile illustrated in Figure 6a. It comprises an anisotropic temporal layer, which has a duration $\Delta t^{\left(2\right)}$ and permittivity $\epsilon^{\left(2\right)}=\text{diag}\left(\epsilon_{X}^{\left(2\right)},\epsilon_{Y}^{\left(2\right)},\epsilon_{Z}^{\left(2\right)}\right)$ , embedded in an isotropic medium with a permittivity $\epsilon^{\left(1\right)}=1$ . The Q matrix of the system can be represented as

(14) $$Q=D_{1}^{-1}D_{2}P_{2}D_{2}^{-1}D_{1}$$

After some mathematical manipulations, we arrive at the following result:

(15) $$t_{pp}=c_{2}^{2}\left(\cos\left(\beta_{x}^{\left(2\right)}\right)+i\alpha_{x}\;\sin\left(\beta_{x}^{\left(2\right)}\right)\right)+s_{2}^{2}\left(\cos\left(\beta_{y}^{\left(2\right)}\right)+i\alpha_{y}\;\sin\left(\beta_{y}^{\left(2\right)}\right)\right)$$

(16) $$t_{ps}=c_{2}s_{2}\left(\cos\left(\beta_{x}^{\left(2\right)}\right)+i\alpha_{x}\;\sin\left(\beta_{x}^{\left(2\right)}\right)-\cos\left(\beta_{y}^{\left(2\right)}\right)+i\alpha_{y}\;\sin\left(\beta_{y}^{\left(2\right)}\right)\right)$$

for which

(17.1) $$\beta_{j}^{\left(2\right)}=\frac{kc\Delta t^{\left(2\right)}}{n_{j}^{\prime \left(2\right)}}=\frac{2\pi f\Delta t^{\left(2\right)}}{n_{j}^{\prime \left(2\right)}}\left(j=x,y\right)$$

(17.2) $$\alpha_{j}=1/2\left(Z_{j}^{\prime \left(2\right)}+1/Z_{j}^{\prime \left(2\right)}\right)\left(j=x,y\right)$$

(17.3) $$c_{2}=\cos\left(\phi \right),s_{2}=\text{sin}\left(\phi \right)$$

where $n_{j}^{\prime \left(2\right)}$ and $Z_{j}^{\prime \left(2\right)}$ have been defined in Eq. (11.3) and (12.3), respectively. Based on these equations, it follows that the property of the anisotropic layer ( $\Delta t^{\left(2\right)},\epsilon^{\left(2\right)}$ ) will solely determine the response of this temporal system to EM waves. Notice that if θ=0 , Eqs. (15) and (16) will reduce to the known results, i.e., Eq. (2) in [24]. In order to better illustrate the polarization conversion effect, let us consider the special case of Complete Polarization Conversion (CPC):

CPC means that the incident wave is completely converted to the orthogonal polarization for certain values of θ , ϕ and frequency, which are denoted as $\theta_{0}$ , $\phi_{0}$ , and $f_{0}$ . Assuming that the incident wave is p -polarized, the CPC condition implies that $t_{pp}\left(\theta_{0},\phi_{0},f_{0}\right)=0$ . Then, we choose values for $\Delta t^{\left(2\right)}$ and $\epsilon^{\left(2\right)}$ that satisfy the following criteria: $\Delta t^{\left(2\right)}=1.11/f_{0},\epsilon^{\left(2\right)}=\text{diag}\left(2.5,12.5,1.25\right)$ . Since $t_{pp}$ is a complicated function of θ ,

ϕ and f , it is convenient to visualize the theoretical results from different perspectives. In Figure 6b, $t_{pp}$ is plotted versus f and θ with $\phi =\phi_{0}$ . While in Figure 6c, $t_{pp}$ is plotted versus f and ϕ with $\theta =\theta_{0}$ . Then in Figure 6d, $t_{pp}$ and $t_{ps}$ are plotted versus f with $\theta =\theta_{0}$ and $\phi =\phi_{0}$ . FDTD simulations were also performed as a validation, and they agree well with the analytical results. From these figures, one can clearly observe that the desired CPC phenomenon is achieved. From this example, we find that, by generalizing to oblique incidence, there is greater flexibility in real-time polarization control compared to what has been achieved in [24].

### 4.3 Anisotropic antireflection temporal coating (AATC)

V. Pacheco-Pena et al. recently introduced the intriguing concept of an antireflection temporal coating (ATC) in the time domain [15]. We know from the theory of temporal boundaries that there is a reflection when the material permittivity undergoes a sudden change (e.g., from $\epsilon^{\left(1\right)}$ to $\epsilon^{\left(1\right)}$ ). However, one can reduce the reflection to zero by ‘inserting’ an intermediate temporal region whose permittivity and duration satisfy the following relations:

$$\epsilon^{\left(2\right)}=\sqrt{\epsilon^{\left(1\right)}\epsilon^{\left(3\right)}}$$

(18) $$\omega_{0}\Delta t^{\left(2\right)}=\left(n-\frac{1}{2}\right)\frac{\pi }{2}$$

where n=1,2,3… and $\omega_{0}$ is the radian frequency of the wave when it is inside the intermediate layer.

In [15], however, the authors only consider the case where the material is isotropic. One may be naturally curious as to what conditions the material properties should satisfy in order to minimize reflection if anisotropic materials are involved. In other words, is it possible to derive a similar expression to Eq. (18) using the GTTMM?

To be more specific, we consider the following temporal structure comprising of three temporal regions, whose permittivities are $\epsilon^{\left(1\right)}$ , $\epsilon^{\left(2\right)}=\left(\epsilon_{X}^{\left(2\right)},\epsilon_{Y}^{\left(2\right)},\epsilon_{Z}^{\left(2\right)}\right),\text{and }\epsilon^{\left(3\right)}$ (see Figure 6a). The second anisotropic layer acts as an anti-reflection temporal coating, which we call an anisotropic antireflection temporal coating (AATC). Our goal is to calculate the permittivity and duration of the AATC, so that there is no net reflection for a certain frequency $f_{0}$ , angle $\theta_{0}$ and $\phi_{0}$ ((i.e., $r_{pp}\left(\theta_{0}.\phi_{0},f_{0}\right)=r_{ps}\left(\theta_{0}.\phi_{0},f_{0}\right)=0$ )), assuming that the incident wave is p- polarized. After some mathematical manipulations (see Supplemental Document 4), we arrive at:

(19.1) $$\frac{c_{1}^{2}}{\epsilon_{X}^{\left(2\right)}}+\frac{s_{1}^{2}}{\epsilon_{Z}^{\left(2\right)}}=\frac{1}{\epsilon_{Y}^{\left(2\right)}}=\frac{1}{\sqrt{\epsilon^{\left(1\right)}\epsilon^{\left(3\right)}}}$$

(19.2) $$\omega_{1}^{\left(2\right)}\Delta t^{\left(2\right)}=\omega_{3}^{\left(2\right)}\Delta t^{\left(2\right)}=\pi /2$$

where the definition of $\omega_{1}^{\left(2\right)}$ and $\omega_{3}^{\left(2\right)}$ have been given in Eq. (11.2). These criteria are easy to interpret: Eq. (19.1) is the impedance matching condition, while Eq. (19.2) represents the π/2 phase accumulation condition. Interestingly, ϕ does not appear in Eq. (19). This means that, as long as the incident angle is θ , there will be no reflection regardless of the orientation of the D and B fields. Moreover, if the wave is normally incident (i.e., $c_{1}=1,s_{1}=0$ ), then Eq. (19) reduces to Eq. (18), assuming $\epsilon_{X}^{\left(2\right)}=\epsilon_{Y}^{\left(2\right)}$ .

**Figure 7:**
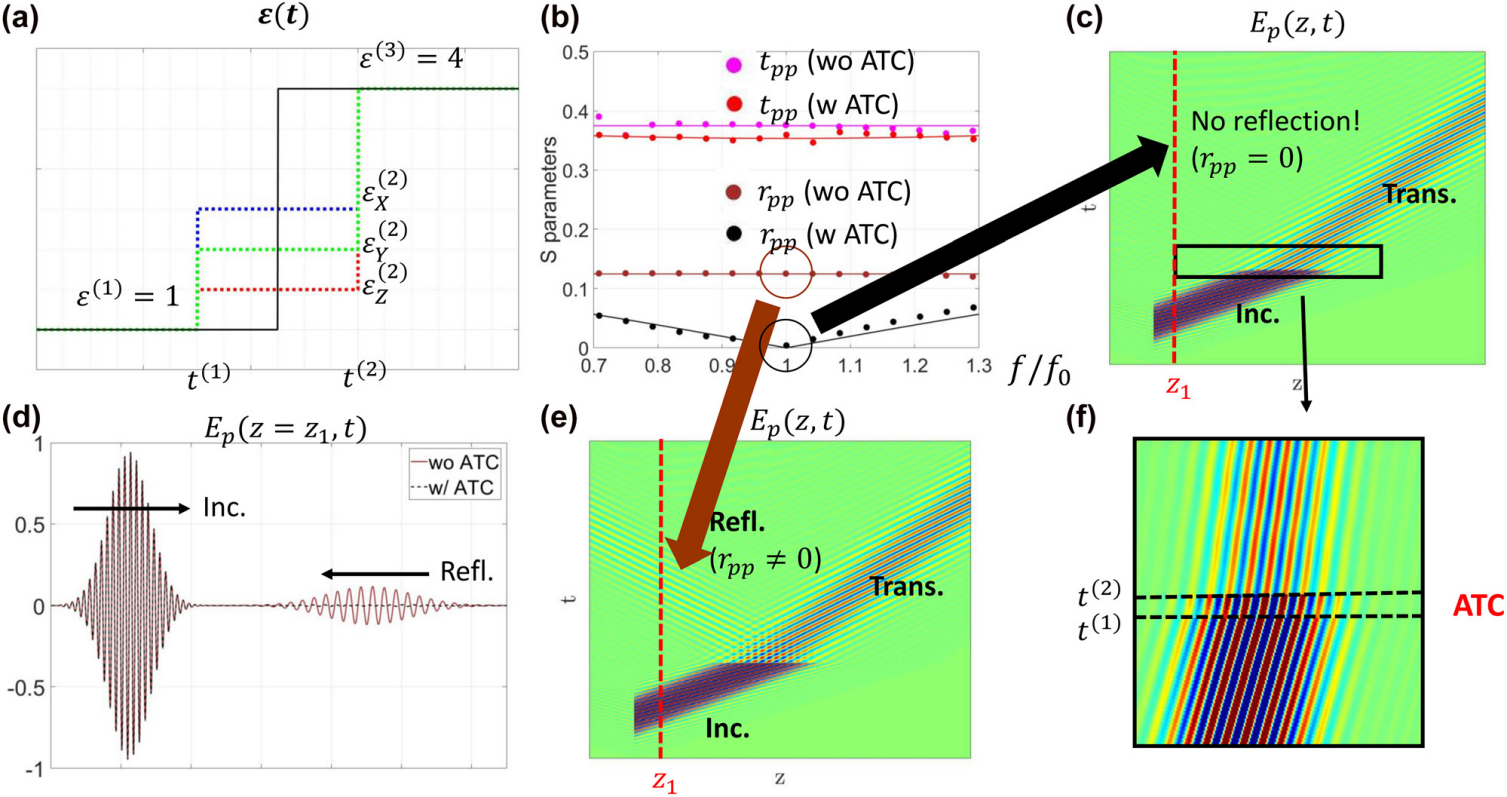
Illustration of an anisotropic anti-reflection temporal coating (AATC). (a) The temporal profiles with and without the AATC. (b) Transmission and reflection spectra with and without the AATC, where the GTTMM and FDTD results are represented by solid lines and dots, respectively. (c) and (e) FDTD simulation results of $E_{p}\left(z,t\right)$ with and without the AATC, at central frequency $f_{0}$ . (f) A magnified plot of a portion of (c), which is indicated by the black rectangle. (d) FDTD simulation results of $E_{p}\left(z=z_{1},t\right)$ with and without the AATC, where the position of $z_{1}$ is indicated by the red dashed lines in (c) and (e).

Next, for a practical illustration, we arbitrarily choose a combination of parameters that satisfy Eq. (19): $\varepsilon^{\left(1\right)}=1,\varepsilon^{\left(2\right)}=\left(1.5,2,3\right),\varepsilon^{\left(3\right)}=4,\Delta t^{\left(2\right)}=\sqrt{2}/4\;T_{0}$ , and $\theta_{0}=\pi /4$ . The temporal profile is depicted by the dotted lines in Figure 7a.

Similar to the approach adopted in Section 4A, we have plotted the spectral response of a p wave propagating in this temporal structure (see Figure 7b). From this figure, we can observe that the reflection ( $r_{pp}$ ) drops to zero at the central frequency $f_{0}=1/T_{0}$ . Notice that the cross-polarization *S* parameters are not presented because $r_{ps}$ is zero across the entire frequency range, if Eq. (19) is satisfied (see a detailed explanation of this phenomenon in Supplemental Document 4). Moreover, we show the real time electric field $E_{p}\left(z,t\right)$ in Figure 7c, where the incident wave is a narrow band Gaussian pulse with a center frequency $f_{0}$ . From this figure, we find that there is no reflection, after the wave meets two temporal boundaries.

As a comparison, we consider another case where ϵ changes suddenly from 1 to four at an instant in time (i.e., the case without an AATC, see the black solid line in Figure 7a). The corresponding *S* parameters and real time electric fields are also plotted in Figure 7b and e. It is important to note that $r_{pp}\neq 0$ at $f=f_{0}$ in this case, which is expected. Besides, in order to better understand the effect of the AATC, we show the electric field distribution at a certain position $z_{1}$ for both with and without AATC cases, in the same plot (Figure 7d). It is seen that the existence of the AATC effectively reduces reflected waves.

This example represents a natural but highly nontrivial generalization of the work reported in [15]. By comparing with the work in [15], our presented results cannot be easily understood from a space-time symmetry perspective. Rather, a rigorous GTTMM analysis is required to derive the conditions for reflection cancellation.

### 4.4 Redirection of energy flow

**Figure 8:**
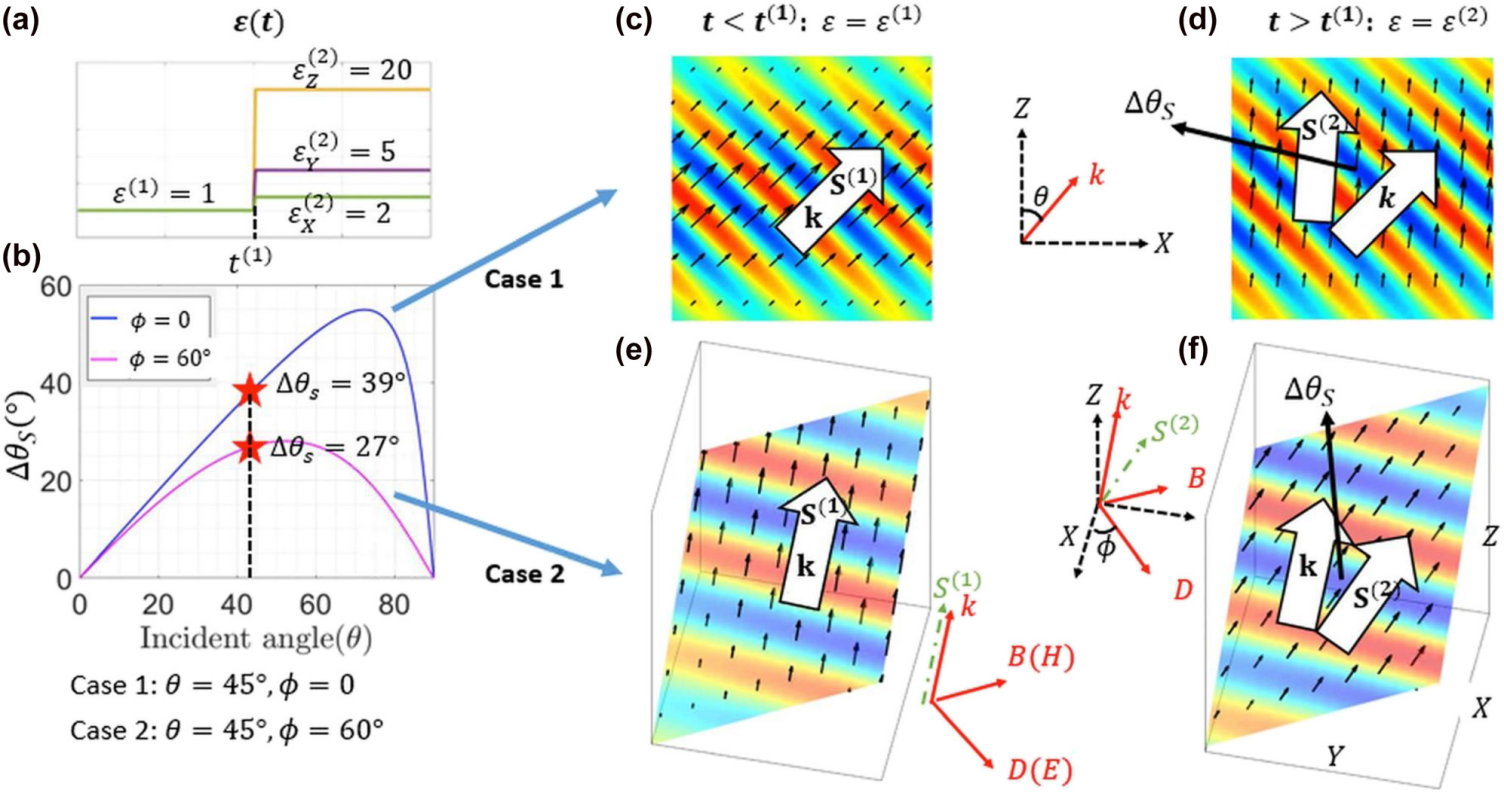
Demonstrations of the redirection of energy propagation. (a) The temporal profile of the system. (b) $\Delta \theta_{S}\text{ as a function}\;\text{of}\;\theta$ for ϕ=0 (blue) and ϕ=60° (magenta), as dictated by Eq. (22). (c)–(f) Normalized $E_{X}$ field (color) and Poynting vector (black arrow) of the incident wave at $=t^{\left(1\right)-}$ , and of the transmitted wave at $t=t^{\left(1\right)+}$ , for the two cases. The spatial positions of the plots are chosen to contain the center of the incident Gaussian beam at the given times. In (e) and (f), the physical quantities ( E and S fields) are plotted on a plane parallel to both $S^{\left(1\right)}$ and $S^{\left(2\right)}$ in order to depict the redirection angle $\Delta \theta_{S}$ between them.

One of the interesting features of temporal boundaries is that the D and B fields are conserved before and after the boundary. This means that the E and H fields will follow the same changes with time as the anisotropy of the material. Hence, the energy flux of the wave (i.e., the Poynting vector), defined as S=E×H , could also change with time. V. Pacheco-Pena et al. investigated this phenomenon in [29] and called it ‘temporal aiming’. The authors of that work, however, only considered a special case when ϕ=0 . One may naturally ask the question: what happens if ϕ≠0? To answer this question, let us first define the temporal system as shown in Figure 8a. The material is isotropic ( $\epsilon^{\left(1\right)}=\mu^{\left(1\right)}=1$ ) in the beginning, and then suddenly switches to an anisotropic form where $\epsilon^{\left(2\right)}=\text{diag}\left(\epsilon_{X}^{\left(2\right)},\epsilon_{Y}^{\left(2\right)},\epsilon_{Z}^{\left(2\right)}\right))$ at $t=t^{\left(1\right)}$ . Here we assume that the material is non-magnetic for simplicity. The orientation of the incident wave is described by the angle θ and ϕ , as depicted in Figure 3b. The Poynting vectors of the incident and transmitted waves (i.e., before and after the temporal boundary) are $S^{\left(1\right)}$ and $S^{\left(2\right)}$ , respectively, and their angle is $\Delta \theta_{S}=<S^{\left(1\right)},S^{\left(2\right)}>$ .

Next, let us calculate the value of $\Delta \theta_{S}$ . Notice that the medium is isotropic before $t^{\left(1\right)}$ , such that $S^{\left(1\right)}$ is parallel to k and $\Delta \theta_{S}=<k,S^{\left(2\right)}>$ . Recalling the coordinate transformation technique introduced in Section 3A (Figure 3c), we have $\Delta S=<z,S^{\left(2\right)}>$ . This means that, if we express $S^{\left(2\right)}$ in x−y−z coordinates, $S^{\left(2\right)}=\left(S_{x}^{\left(2\right)},S_{y}^{\left(2\right)},S_{z}^{\left(2\right)}\right)$ , then $\Delta \theta_{S}$ can be easily calculated using the following expression:

(20) $$\Delta \theta_{S}=\cos^{-1}\left(\frac{S_{z}^{\left(2\right)}}{\left|S^{\left(2\right)}\right|}\right)$$

where $S^{\left(2\right)}=E^{\left(2\right)+}\times H^{\left(2\right)+}$ . Notice that the ‘+’ sign in the superscript denotes the transmitted wave. The transmission coefficients can be computed using the Q matrix of the system:

(21) $$Q=D_{1}^{-1}D_{2}=M_{12}$$

After simplification (see Supplemental Document 3), we have

(22) $$\Delta \theta_{S}=\tan^{-1}\left(\frac{c_{1}c_{2}s_{1}\left(1/\;\epsilon_{X}^{\left(2\right)}-1/\epsilon_{Z}^{\left(2\right)}\right)}{c_{2}^{2}\left(c_{1}^{2}/\epsilon_{X}^{\left(2\right)}+s_{1}^{2}/\epsilon_{Z}^{\left(2\right)}\right)+s_{2}^{2}/\epsilon_{Y}^{\left(2\right)}}\right)$$

Clearly, $\Delta \theta_{S}$ is a function of both the material anisotropy ( $\epsilon^{\left(2\right)}$ ), and the orientation of the incident wave ( θ,ϕ ). It is interesting to observe that if ϕ=0 , then Eq. (22) will reduce to $\Delta \theta_{\text{S}}=\tan^{-1}\left(\tan\left(\theta \right)\cdot \epsilon_{X}^{\left(2\right)}/\epsilon_{Z}^{\left(2\right)}\right),$ which is in agreement with Eq. (2) in [29].

Now, we consider a specific material profile: $\epsilon^{\left(2\right)}=\text{diag}\left(2,5,20\right),$ which is schematically illustrated in Figure 8a. Using Eq. (22), $\Delta \theta_{S}$ is plotted against θ for ϕ=0 and ϕ=60° in Figure 8b. Following this figure, we study two cases: (1) θ=45°,ϕ=0, and (2) θ=45°,ϕ=60°, which correspond to $\Delta \theta_{S}=39{}^{\circ}$ and 27° , respectively. In Figure 8c–f, we present overlapping plots of the electric fields and the instantaneous Poynting vectors for the two cases just before and after the temporal boundary (at $t=t^{\left(1\right)+}$ and $t=t^{\left(1\right)-}$ ). We observe that the normal of the phase front ( k ) and Poynting vector ( S ) are along the same direction at $t=t^{\left(1\right)-}$ , while they have a nonzero angle at $t=t^{\left(1\right)+}$ . Using the simulated data from lumerical FDTD, we confirm that this angle matches very well the theoretical predications in Figure 8b. Moreover, it is interesting to note that in case 1, $S^{\left(1\right)}$ and $S^{\left(2\right)}$ lie in the XZ plane, therefore, Figure 8c–d are rendered in 2D. For case 2, however, $S^{\left(1\right)}$ and $S^{\left(2\right)}$ are not along any specific axial direction and, therefore, the results in Figure 8e and f are displayed on a plane in 3D space. From the results, one can clearly see that ϕ≠0 represents a highly nontrivial scenario, which includes the special case of ϕ=0 considered in [29].

## 5 Conclusions

In this paper, we proposed a rigorous analytical methodology, which we call GTTMM, to evaluate wave propagation in temporally stratified structures, based on the application of the appropriate boundary conditions to Maxwell’s equations. Then we applied this theory to several temporal systems, and confirmed its validity using full-wave FDTD simulations. These studies targeted anisotropic material systems, which have important applications but are typically difficult to solve due to their relatively complex mathematical descriptions. Comparing with the traditional methods presented in [13, 15, 16, 18, 29], our approach is more elegant as well as concise. With this tool, we can tackle more complicated problems; all four TBVPs we considered represent generalizations of previously studied system. More importantly, it is universal and can be applied as a powerful tool for solving a very broad class of TBVPs. From antireflection coatings to polarizers, we have shown that these completely different applications can be considered as part of the same overarching theoretical framework. In addition to the effectiveness demonstrated in solving these problems, this framework also reveals some rich insights into the fundamental physics. First, it reveals the mathematical similarities between all these seemingly different systems. Besides, it sheds light on the unique properties of temporal boundaries and has prompted us to reconsider some well-established concepts such as oblique incidence. In conclusion, our formalism represents a powerful tool for solving TBVPs. Moreover, it is expected to serve an important role in the future design of potentially transformative devices, as the current temporal modulation techniques continue to grow more mature.

## Supplementary Material

Supplementary Material
