# Supplementary material for: Generalized temporal transfer matrix method: a systematic approach to solving electromagnetic wave scattering in temporally stratified structures
Source: Nanophotonics. 2022 Mar 7;11(7):1309–20. doi: 10.1515/nanoph-2021-0715 (PMC11501795; doi:10.1515/nanoph-2021-0715)
Supplement: Supplementary file 1 — Supplementary Material [file j_nanoph-2021-0715_suppl.docx]

Supplementary Document

**1. Derivation of the dispersion relation in Section 3B**

In Section 3B of the main text, we introduce the dispersion relation ($\boldsymbol{k}\sim\omega_{\sigma}^{\left( i \right)}(\sigma=1,2,3,4)$) for anisotropic temporal systems (see Eq. (11.1) and Eq. (11.2)). Here, we provide a thorough derivation of this relation and the corresponding choice of the vector $\boldsymbol{d}$. The discussion is divided into two subsections, depending on whether the temporal layer is anisotropic.

**A. General case: anisotropic temporal layer**

In this subsection, we derive the dispersion relation for electromagnetic (EM) waves propagating in an anisotropic medium, at an oblique incidence angle (see Eq. (11.1) and (11.2) in the main text).

First, in the S3 coordinate system, the electric displacement $\boldsymbol{D}=(D_{x}, D_{y}, 0)$ and the wave vector $\boldsymbol{k}=(0,0,k)$ should satisfy the following equation (Eq. (5) in the main text):

| $\boldsymbol{k}\times\left[ \eta\left( \boldsymbol{k}\boldsymbol{\times}\left( \delta\boldsymbol{D} \right) \right) \right]=-\frac{\omega^{2}}{c^{2}}\boldsymbol{D}$ | (S1) |
| --- | --- |

where we define $\delta=\varepsilon^{-1}, \eta=\mu^{-1}$. Using Eqs. (4.1)-(4.3) in the main text, we can express the explicit forms of $\delta$ and $\eta$ as:

|  | $\delta\boldsymbol{=}\frac{1}{\varepsilon_{X}\varepsilon_{Y}\varepsilon_{Z}}\left( \begin{matrix} \varepsilon_{Y}(c_{1}^{2}\varepsilon_{Z}+s_{1}^{2}\varepsilon_{X}) & 0 & -c_{1}s_{1}\varepsilon_{Y}(\varepsilon_{Z}-\varepsilon_{X}) \\ 0 & \varepsilon_{X}\varepsilon_{Z} & 0 \\ -c_{1}s_{1}\varepsilon_{Y}(\varepsilon_{Z}-\varepsilon_{X}) & 0 & \varepsilon_{Y}(c_{1}^{2}\varepsilon_{X}+s_{1}^{2}\varepsilon_{Z}) \end{matrix} \right)$  $\eta\boldsymbol{=}\frac{1}{\mu_{X}\mu_{Y}\mu_{Z}}\left( \begin{matrix} \mu_{Y}(c_{1}^{2}\mu_{Z}+s_{1}^{2}\mu_{X}) & 0 & -c_{1}s_{1}\mu_{Y}(\mu_{Z}-\mu_{X}) \\ 0 & \mu_{X}\mu_{Z} & 0 \\ -c_{1}s_{1}\mu_{Y}(\mu_{Z}-\mu_{X}) & 0 & \mu_{Y}(c_{1}^{2}\mu_{X}+s_{1}^{2}\mu_{Z}) \end{matrix} \right)$ | (S2) |
| --- | --- | --- |

Note that, in order to simplify the notation, we do not use superscripts for permittivity and permeability as has been done in the main text.

At this point, we evaluate the left side of Eq. (S1) by using the following step-by-step procedure:

|  | $\delta\boldsymbol{D}=\left( \delta_{xx}D_{x}, \delta_{yy}D_{y}, \delta_{zx}D_{x} \right)$  $\boldsymbol{k}\times\delta\boldsymbol{D}\mathbf{=}k\left( -\delta_{yy}D_{y}, \delta_{xx}D_{x}, 0 \right)$  $\eta\left( \boldsymbol{k}\times\delta\boldsymbol{D} \right)\mathbf{=}k\left( -\delta_{yy}\eta_{xx}D_{y}, \delta_{xx}{\eta_{yy}D}_{x}, -\delta_{yy}\eta_{zx}D_{y} \right)$  $\boldsymbol{k}\times[\eta\left( \boldsymbol{k}\times\delta\boldsymbol{D} \right)\boldsymbol{]=}k^{2}(-\delta_{xx}\eta_{yy}D_{x}, -\delta_{yy}\eta_{xx}D_{y}, 0)$ | (S3) |
| --- | --- | --- |

Next, by substituting the result in the last step of the above equation into Eq. (S1), we have

|  | $k^{2}\left( -\delta_{xx}\eta_{yy}D_{x}, -\delta_{yy}\eta_{xx}D_{y}, 0 \right)+\frac{\omega^{2}}{c^{2}}\left( D_{x}, D_{y}, 0 \right)=0$ | (S4) |
| --- | --- | --- |

Rewriting Eq. (S4) in matrix form yields:

|  | $\left( \begin{matrix} \frac{\omega^{2}}{c^{2}}-k^{2}\delta_{yy}\eta_{xx} & 0 \\ 0 & \frac{\omega^{2}}{c^{2}}-k^{2}\delta_{xx}\eta_{yy} \end{matrix} \right)\left( \begin{matrix} D_{y} \\ D_{x} \end{matrix} \right)=\tilde{F}\tilde{D}=0,$ | | | (S5) |
| --- | --- | --- | --- | --- |
|  | |  |  |  |

In order to have a nontrivial solution of $\boldsymbol{D}$, the determinant of the matrix $\tilde{F}$ defined in Eq. (S5) should be zero. Namely, the following conditions must be satisfied:

|  | $\frac{\omega^{2}}{c^{2}}-k^{2}\delta_{yy}\eta_{xx}=0$ | (S6.1) |
| --- | --- | --- |
|  | $\frac{\omega^{2}}{c^{2}}-k^{2}\delta_{xx}\eta_{yy}=0$ | (S6.2) |

and the corresponding eigenvectors of $\tilde{D}=\left( D_{y},D_{x} \right)^{T}$are $\left( 1,0 \right)^{T}$ and $\left( 0,1 \right)^{T}$.

By solving Eq. (S6), we can retrieve Eq. (11.1) and (11.2) in the main text, if we let $\omega_{1}=-\omega_{2}$ be the solution of Eq. (S6.1), and $\omega_{3}=-\omega_{4}$ be the solution of Eq. (S6.2). Then, by comparing this result with Eq. (6.2) in the main text, we conclude that

|  | $\boldsymbol{d}_{\boldsymbol{1}}^{\left( \boldsymbol{i} \right)}=\boldsymbol{d}_{\boldsymbol{2}}^{\left( \boldsymbol{i} \right)}=\left( 0,1,0 \right), \boldsymbol{d}_{\boldsymbol{3}}^{\left( \boldsymbol{i} \right)}=\boldsymbol{d}_{\boldsymbol{4}}^{\left( \boldsymbol{i} \right)}=(1,0,0)$ | (S7) |
| --- | --- | --- |

which is in agreement with Eq. (11.4) in the main text.

**B. Special case: isotropic temporal layer**

It is expected that the discussion in Section 1A should include as a special case the scenario in which the material is isotropic. In this case, $\varepsilon^{(i)}$ and $\mu^{(i)}$ defined in Eq. (4.2) in the main text are reduced from tensors to scalar quantities. Let us define $\varepsilon=\varepsilon_{X}=\varepsilon_{Y}=\varepsilon_{Z}$ and $\mu=\mu_{X}=\mu_{Y}=\mu_{Z}$, then under these conditions Eq. (S5) reduces to:

|  | $\left( \begin{matrix} \frac{\omega^{2}}{c^{2}}-\frac{k^{2}}{\varepsilon\mu} & 0 \\ 0 & \frac{\omega^{2}}{c^{2}}-\frac{k^{2}}{\varepsilon\mu} \end{matrix} \right)\left( \begin{matrix} D_{y} \\ D_{x} \end{matrix} \right)=\tilde{F}\tilde{D}=0$ | (S8) |
| --- | --- | --- |

Notice that the matrix $\tilde{F}$ in the above equation becomes an identity matrix. So, as long as the condition

|  | $\omega^{2}/c^{2}=k^{2}/\varepsilon\mu$ | (S9) |
| --- | --- | --- |

is satisfied, Eq. (S8) will always hold, regardless of the choice of $D_{y}$ and $D_{x}$ (from a mathematical perspective, the eigenvector of an $n\times n$ identity matrix is any vector). In other words, we now have the freedom to choose the base vectors in Eq. (11.4) in the main text. Therefore, we let

|  | $\boldsymbol{d}_{\boldsymbol{1}}^{\left( \boldsymbol{i} \right)}=\boldsymbol{d}_{\boldsymbol{2}}^{\left( \boldsymbol{i} \right)}=\left( -sin(\phi),\cos(\phi),0 \right), \boldsymbol{d}_{\boldsymbol{3}}^{\left( \boldsymbol{i} \right)}=\boldsymbol{d}_{\boldsymbol{4}}^{\left( \boldsymbol{i} \right)}=(\cos(\phi),\sin(\phi),0)$ | (S10) |
| --- | --- | --- |

which corresponds to the forward and backward propagating modes of $s-$ and $p-$ polarized waves (see the schematic diagrams illustrated in Fig. S1).

Next, substituting Eq. (S10) into Eq. (10) in the main text, we arrive at

| $\mathcal{D}_{i}=$ $\left( \begin{matrix} \mathbf{cos}\boldsymbol{(\phi)} & \mathbf{cos}\boldsymbol{(\phi)} & \mathbf{sin}\boldsymbol{(\phi)} & \mathbf{sin}\boldsymbol{(\phi)} \\ \boldsymbol{-}\mathbf{cos}\left( \boldsymbol{\phi} \right)\boldsymbol{Z}^{\boldsymbol{(i)}} & \mathbf{cos}\left( \boldsymbol{\phi} \right)\boldsymbol{Z}^{\boldsymbol{(i)}} & \boldsymbol{-}\mathbf{sin}\left( \boldsymbol{\phi} \right)\boldsymbol{Z}^{\boldsymbol{(i)}} & \mathbf{sin}\left( \boldsymbol{\phi} \right)\boldsymbol{Z}^{\boldsymbol{(i)}} \\ \mathbf{-sin}\left( \boldsymbol{\phi} \right)\boldsymbol{Z}^{\boldsymbol{(i)}} & \mathbf{sin}\left( \boldsymbol{\phi} \right)\boldsymbol{Z}^{\boldsymbol{(i)}} & \mathbf{cos}\left( \boldsymbol{\phi} \right)\boldsymbol{Z}^{\boldsymbol{(i)}} & \mathbf{-cos}\left( \boldsymbol{\phi} \right)\boldsymbol{Z}^{\boldsymbol{(i)}} \\ \boldsymbol{-}\mathbf{sin}\boldsymbol{(\phi)} & \boldsymbol{-}\mathbf{sin}\boldsymbol{(\phi)} & \mathbf{cos}\boldsymbol{(\phi)} & \mathbf{cos}\boldsymbol{(\phi)} \end{matrix} \right)$ | (S11) |
| --- | --- |

where $Z^{(i)}=\sqrt{\mu^{(i)}/\varepsilon^{(i)}}$is the impedance of the $i$-th temporal layer, normalized to $Z_{0}$.

For the first three of the four TBVPs considered in Section 4 of the main text, the first and last layers of the corresponding temporal structure are isotropic, where the expressions for $\mathcal{D}$ correspond to Eq. (S11).


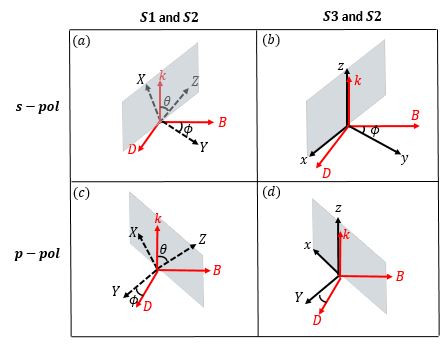


Fig. S1. Geometric illustration of the two polarization states based on the (a)(c) S1 and S2, and (b)(d) S3 and S2 coordinate systems. Notice that (a)(c) are identical to Fig. 5(a) in the main text.

**2. Applications of GTTMM in isotropic systems**

In this section, we will demonstrate how the GTTMM can be applied to two TBVPs that have recently been reported, namely, effective media [1] and photonic crystals [2]. Note that the two cases considered here only involve isotropic materials.

When the materials are isotropic (*i.e*., all temporal layers are isotropic), there is no need to distinguish between $s-$ and $p-$ polarized waves, and the $4\times4$ matrix formalism would be redundant. One can easily prove that the transfer matrices ($\mathcal{D,}P$) will reduce to the following $2\times2$ forms:

|  | $\mathcal{D}_{i}=\frac{1}{2}\left( \begin{matrix} 1 & 1 \\ -{Z_{0}Z}^{(i)} & Z_{0}Z^{(i)} \end{matrix} \right)$  $P_{i}=\left( \begin{matrix} e^{i\omega^{(i)}\Delta t^{(i)}} & \\ & e^{-i\omega^{(i)}\Delta t^{(i)}} \end{matrix} \right)$ | (S12) |
| --- | --- | --- |

where $\omega^{\left( i \right)}=ck/\sqrt{\varepsilon^{(i)}\mu^{(i)}}$ is the natural definition of the dispersion relation according to Eq. (S9).


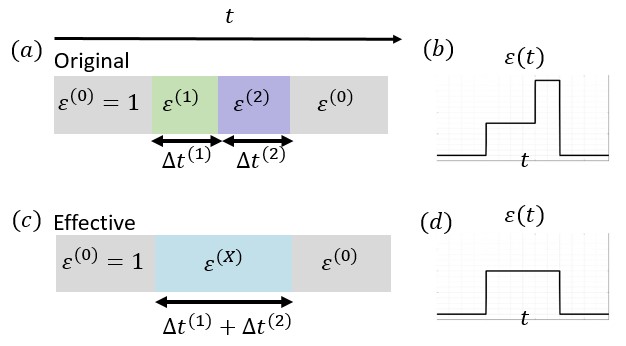
**A. Effective medium**

Fig. S2. Schematics illustrating the derivation of an effective medium using GTTMM. (a),(b) Structure 1: The unit cell of the 1-2-1-2 structure embedded in a background medium ($\varepsilon^{(0)}=Z^{\left( 0 \right)}=1$). (c),(d) Structure 2: The effective medium embedded in a background medium.

Fig. S1. Schematics illustrating the derivation of an effective medium using GTTMM. (a),(b) Structure 1: The unit cell of the 1-2-1-2 structure embedded in a background medium ($\varepsilon^{(0)}=Z^{\left( 0 \right)}=1$). (c),(d) Structure 2: The effective medium embedded in a background medium.

In the field of electromagnetics, the concept of an effective medium [1] is extremely important. In [3], V. Pacheco-Pena *et al*. extended this notion to the time domain, where a wave travels in a material whose permittivity temporally alternates between $\varepsilon^{(1)}$and $\varepsilon^{(2)}$, with a period much smaller than that of the incident wave. This is referred to as the ‘1-2-1-2 structure’. The objective is to find a medium with an effective permittivity $\varepsilon^{(x)}$, which is equivalent to this 1-2-1-2 structure. In order to derive the expression for $\varepsilon^{(x)}$, let us consider the two temporal structures as depicted in Fig. S2(a),(b) and (c),(d) respectively. It is obvious that the $Q$ matrices of the two systems are given by

|  | $Q=M_{01}P_{1}M_{12}P_{2}M_{20}$  $Q^{eff}=M_{0X}P_{X}M_{X0}$ | (S13) |
| --- | --- | --- |

By letting $Q=Q^{eff}$, and assuming that $\Delta t^{(1)}, \Delta t^{(2)}\to0$ (see Section 2C of this document for details of this derivation), we find that

|  | $\alpha_{01}\Delta\phi^{(1)}+\alpha_{02}\Delta\phi^{(2)}=\alpha_{0X}\Delta\phi^{(x)}$ | (S14) |
| --- | --- | --- |

where the two parameters $\alpha$ and $\Delta\phi^{(i)}$ are defined as

|  | $\alpha_{ij}=Z^{(i)}/Z^{(j)}+Z^{(j)}/Z^{(i)}$, $\Delta\phi^{(i)}=\frac{ck}{\sqrt{\varepsilon^{(i)}\mu^{(i)}}}\Delta t^{(i)}$ | (S15) |
| --- | --- | --- |

After simplification, we have

|  | $\varepsilon^{(x)}=\frac{\varepsilon^{\left( 1 \right)}\varepsilon^{\left( 2 \right)}(\Delta t^{\left( 1 \right)}+\Delta t^{(2)})}{\varepsilon^{(1)}\Delta t^{(2)}+\varepsilon^{(2)}\Delta t^{(1)}}$ | (S16) |
| --- | --- | --- |

which agrees with Eq. (7e) in [3].

From a physical perspective, this equation suggests that the inverse of the effective permittivity ($1/\varepsilon^{(x)}$) is the weighted arithmetic mean of $1/\varepsilon^{(1)}$ and $1/\varepsilon^{(2)}$ , where the weights are the durations of the two corresponding periods.

We note that it is instructive to compare this result with its spatial counterpart. In other words, a structure composed of two materials which are stacked in an alternating and periodic fashion. From conventional effective medium theory, it follows that the effective permittivity can be written as:

|  | $\varepsilon^{(x)}=\frac{\varepsilon^{(1)}\Delta x^{(1)}+\varepsilon^{(2)}\Delta x^{(2)}}{\Delta x^{(1)}+\Delta x^{(2)}}$ | (S17) |
| --- | --- | --- |

which means that $\varepsilon^{(x)}$ is the weighted arithmetic mean of $\varepsilon^{(1)}$ and $\varepsilon^{(2)}$, where the weight is the thickness of the two constituent materials.

**B. Photonic crystals**


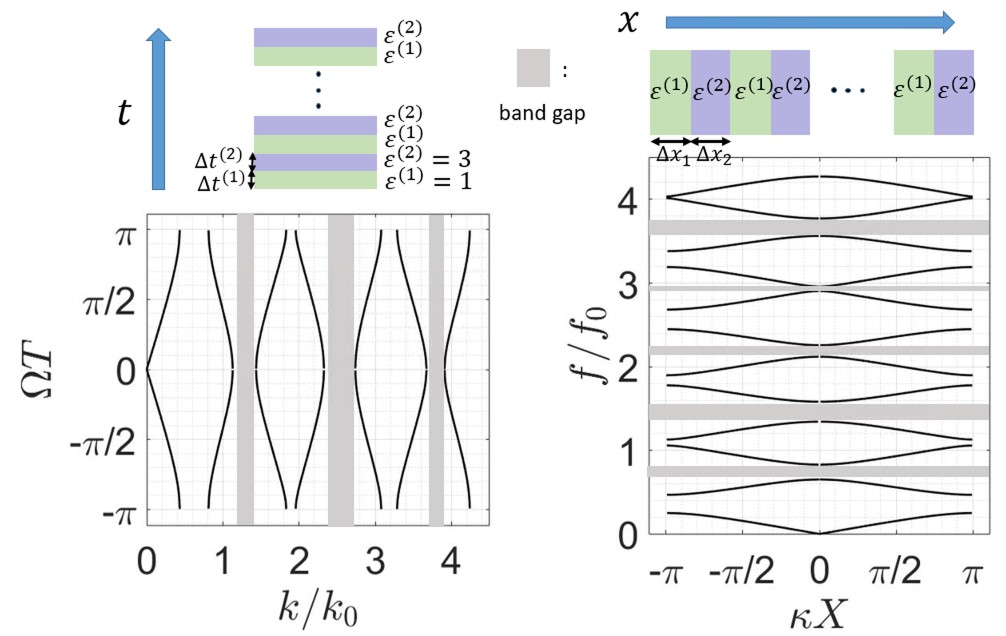


Fig. S3. The profile (a)-(b) and dispersion relation (c)-(d) of a time domain photonic crystal and its traditional counterpart. The grey shaded areas indicate the existence of bandgaps.

Photonic crystals (PCs) have been an extensively researched topic since they were first proposed in [4]. The most interesting and important property of PCs is their frequency bandgap, which means that an incident wave with a certain frequency cannot pass through the crystal. Segev *et al*. conceived the idea of a photonic time-crystal (PTC) [2]. The temporal profile of the PTC is the same as that described in the previous subsection. From one perspective, the transfer matrix of the unit cell (1-2-1-2 structure) can be written straightforwardly as:

|  | $Q=M_{12}P_{2}M_{21}P_{1}$ | (S18) |
| --- | --- | --- |

From the other perspective, we enforce periodicity constraints on the unit cell of the photonic crystal:

|  | $D^{+}\left( t+T \right)=D^{+}(t)\cdot exp(i\Omega T)$,  $D^{-}\left( t+T \right)=D^{-}(t)\cdot exp(-i\Omega T)$, | (S19) |
| --- | --- | --- |

where $T=\Delta t^{\left( 1 \right)}+\Delta t^{\left( 2 \right)}$, and the superscripts ‘+’ and ‘-’ denote waves propagating in forward and backward directions respectively. Hence, if we rewrite Eq. (S19) in matrix form:

|  | $Q^{\mathrm{PC}}=\left( \begin{matrix} exp(i\Omega T) & 0 \\ 0 & exp(-i\Omega T) \end{matrix} \right)$ | (S20) |
| --- | --- | --- |

then, after employing Eq. (S18), we have

|  | $\mathrm{Tr}\left( Q \right)=Q_{11}+Q_{22}=2\cos\left( \Delta\phi^{\left( 1 \right)}+\Delta\phi^{\left( 2 \right)} \right)$  $-(\alpha_{12}-2)sin(\Delta\phi^{\left( 1 \right)})sin(\Delta\phi^{\left( 2 \right)})$ | (S21) |
| --- | --- | --- |

where the definition of $\Delta\phi$ and $\alpha$ has been given in Eq. (S15).

In the end, these two perspectives should yield results that are equivalent to each other, *i.e.* $\mathrm{Tr}\left( Q \right)=\mathrm{Tr}(Q^{PC})$. Hence, we have:

|  | $\cos\left( \Omega T \right)=\cos\left( ck\sum_{i=1}^{2} \left( \Delta t^{\left( i \right)}/n^{(i)} \right) \right)$  $-\frac{1}{2}(\alpha_{12}-2)sin(\Delta\phi^{\left( 1 \right)})sin(\Delta\phi^{(2)})$ | (S22) |
| --- | --- | --- |

Solving this equation, one can obtain the dispersion relation of a photonic time crystal $\Omega\sim k$.

From a knowledge of traditional PCs, the dispersion relation $\kappa\sim\omega$ can be extracted from the following equation [5]:

|  | $\cos\left( \kappa X \right)=\cos\left( \frac{\omega}{c}\sum_{i=1}^{2} \left( n^{(i)}\Delta x^{(i)} \right) \right)$  $-\frac{1}{2}(\alpha_{12}-2)sin(\Delta\phi^{\left( 1 \right)})sin(\Delta\phi^{(2)})$ | (S23) |
| --- | --- | --- |

where $\omega$ is the incident wave frequency, $\Delta x^{(1)}$ and $\Delta x^{(2)}$ are the thicknesses of the two constituent materials, $X=\Delta x^{(1)}+\Delta x^{(2)}$ is the spatial period, and $\Delta\phi^{(i)}=n^{(i)}\Delta x^{(i)}\omega/c (i=1,2)$ are the propagation phases within the two materials. Solving Eq. (S23) results in the dispersion relation for a traditional (spatial) photonic crystal $\kappa\sim\omega$.

In order to better understand the relationship between temporal and spatial photonic crystals, we plot the dispersion relation $\Omega\sim k$ and $\kappa\sim\omega$ dictated by Eq. (S22) and (S23) in Fig. S3(a) and (b), respectively. For simplicity, we let $\Delta t^{\left( 1 \right)}=\Delta t^{\left( 2 \right)}$ and $\Delta x^{\left( 1 \right)}=\Delta x^{\left( 2 \right)}$. Moreover, $\varepsilon^{(1)}=1, \varepsilon^{(2)}=3$ is assumed for both cases. From Fig. S3, one can easily recognize the duality between space and time. To this end, the traditional PC has frequency bandgaps, while the PTC has momentum bandgaps.

**C. Derivation of an important identity in Section 2A (effective medium)**

In Section 2A of this document where we discussed the concept of a temporal effective medium, an important identity is given without proof (Eq. (S14)):

|  | $\alpha_{01}\Delta\phi^{(1)}+\alpha_{02}\Delta\phi^{(2)}=\alpha_{0X}\Delta\phi^{(x)}$ | (S24) |
| --- | --- | --- |

Here we will prove this identity by developing the explicit expressions for $Q$ and $Q^{eff}$ (decided by Eq. (S13))

**C.1 Expression for the** $\boldsymbol{Q}$ **matrix**

First, we will calculate the explicit expression for $Q$ as follows

|  | $Q=M_{01}P_{1}M_{12}P_{2}M_{20}$  $=\frac{1}{2}\left( \begin{matrix} t_{1} & r_{1} \\ r_{1} & t_{1} \end{matrix} \right)\left( \begin{matrix} e^{i\Delta\phi^{\left( 1 \right)}} & 0 \\ 0 & e^{-i\Delta\phi^{\left( 1 \right)}} \end{matrix} \right)\frac{1}{2}\left( \begin{matrix} t_{2} & r_{2} \\ r_{2} & t_{2} \end{matrix} \right)\left( \begin{matrix} e^{i\Delta\phi^{\left( 2 \right)}} & 0 \\ 0 & e^{-i\Delta\phi^{\left( 2 \right)}} \end{matrix} \right)\frac{1}{2}\left( \begin{matrix} t_{0} & r_{0} \\ r_{0} & t_{0} \end{matrix} \right)$ | (S25) |
| --- | --- | --- |

where $t_{0}, r_{0},t_{1},r_{1},t_{2},r_{2}$ are equal to $1+\frac{Z^{\left( 2 \right)}}{Z^{(0)}},1-\frac{Z^{\left( 2 \right)}}{Z^{\left( 0 \right)}},1+\frac{Z^{\left( 0 \right)}}{Z^{\left( 1 \right)}},1-\frac{Z^{\left( 0 \right)}}{Z^{\left( 1 \right)}},1+\frac{Z^{\left( 1 \right)}}{Z^{\left( 2 \right)}},1-\frac{Z^{\left( 1 \right)}}{Z^{(2)}}$, respectively.

Then, upon further simplification, we find that

|  | Q$=\frac{1}{8}\left( \begin{matrix} t_{1}e^{i\Delta\phi^{\left( 1 \right)}} & r_{1}e^{-i\Delta\phi^{\left( 1 \right)}} \\ r_{1}e^{i\Delta\phi^{\left( 1 \right)}} & t_{1}e^{-i\Delta\phi^{\left( 1 \right)}} \end{matrix} \right)\left( \begin{matrix} t_{2}e^{i\Delta\phi^{\left( 2 \right)}} & r_{2}e^{-i\Delta\phi^{\left( 2 \right)}} \\ r_{2}e^{i\Delta\phi^{\left( 2 \right)}} & t_{2}e^{-i\Delta\phi^{\left( 2 \right)}} \end{matrix} \right) \left( \begin{matrix} t_{0} & r_{0} \\ r_{0} & t_{0} \end{matrix} \right)$ $=\left( \begin{matrix} Q_{11} & Q_{12} \\ Q_{21} & Q_{22} \end{matrix} \right)$ | (S26) |
| --- | --- | --- |

First, let us consider the term $Q_{11}$, which is one of the matrix elements of $Q$:

|  | $Q_{11}=[t_{0}t_{1}t_{2}e^{i\left( \Delta\phi^{\left( 1 \right)}+\Delta\phi^{\left( 2 \right)} \right)}+t_{0}r_{1}r_{2}e^{i\left( -\Delta\phi^{\left( 1 \right)}+\Delta\phi^{\left( 2 \right)} \right)}+r_{0}t_{1}r_{2}e^{i\left( \Delta\phi^{\left( 1 \right)}-\Delta\phi^{\left( 2 \right)} \right)}+r_{0}r_{1}t_{2}e^{i\left( -\Delta\phi^{\left( 1 \right)}-\Delta\phi^{\left( 2 \right)} \right)}]/8$ | (S27) |
| --- | --- | --- |

where $t_{0}t_{1}t_{2}=(1+\frac{Z^{\left( 2 \right)}}{Z^{(0)}}$)$(1+\frac{Z^{\left( 0 \right)}}{Z^{(1)}}$)$\left( 1+\frac{Z^{\left( 1 \right)}}{Z^{\left( 2 \right)}} \right)=2+\alpha_{01}+\alpha_{12}+\alpha_{02}$

Similarly, we have

|  | $t_{0}r_{1}r_{2}=2-\alpha_{01}-\alpha_{12}+\alpha_{02}$  $r_{0}t_{1}r_{2}=2+\alpha_{01}-\alpha_{12}-\alpha_{02}$  $r_{0}r_{1}t_{2}=2-\alpha_{01}+\alpha_{12}-\alpha_{02}$ | (S28) |
| --- | --- | --- |

Then, considering $\Delta\phi^{\left( 1 \right)}\to0$ and $\Delta\phi^{\left( 2 \right)}\to0$, $Q_{11}$ can be expressed as:

|  | $Q_{11}=\left[ \left( 2+\alpha_{01}+\alpha_{12}+\alpha_{20} \right)\left( 1+\Delta\phi^{\left( 1 \right)}+\Delta\phi^{\left( 2 \right)} \right)+\left( 2-\alpha_{01}-\alpha_{12}+\alpha_{02} \right)\left( 1-\Delta\phi^{\left( 1 \right)}+\Delta\phi^{\left( 2 \right)} \right)+\left( 2+\alpha_{01}-\alpha_{12}-\alpha_{02} \right)\left( 1+\Delta\phi^{\left( 1 \right)}-\Delta\phi^{\left( 2 \right)} \right)+\left( 2-\alpha_{01}+\alpha_{12}-\alpha_{02} \right)\left( 1-\Delta\phi^{\left( 1 \right)}-\Delta\phi^{\left( 2 \right)} \right) \right]/8$ | (S29.1) |
| --- | --- | --- |

After simplification, we arrive at the result

|  | $Q_{11}=1+\frac{1}{2}\alpha_{01}\Delta\phi^{(1)}+\frac{1}{2}\alpha_{02}\Delta\phi^{(2)}$ | (S29.2) |
| --- | --- | --- |

**C.2 Expression for the** $\boldsymbol{Q}^{\boldsymbol{eff}}$ **matrix**

Next, we will evaluate $Q^{eff}$ in the same manner by starting with the expression

|  | $Q^{eff}=M_{0X}P_{X}M_{X0}$  $=\frac{1}{2}\left( \begin{matrix} t_{x} & r_{x} \\ r_{x} & t_{x} \end{matrix} \right)\left( \begin{matrix} e^{i\Delta\phi^{\left( x \right)}} & 0 \\ 0 & e^{-i\Delta\phi^{\left( x \right)}} \end{matrix} \right)\frac{1}{2}\left( \begin{matrix} t_{x}^{'} & r_{x}^{'} \\ r_{x}^{'} & t_{x}^{'} \end{matrix} \right)$ | (S30) |
| --- | --- | --- |

where $t_{x}, r_{x}, t_{x}^{'},r_{x}^{'}$ are equal to $1+\frac{Z^{\left( 0 \right)}}{Z^{(x)}},1-\frac{Z^{\left( 0 \right)}}{Z^{\left( x \right)}},1+\frac{Z^{\left( x \right)}}{Z^{\left( 0 \right)}},1-\frac{Z^{\left( x \right)}}{Z^{\left( 0 \right)}}$, respectively.

Hence,

|  | $Q^{eff}=\frac{1}{4}\left( \begin{matrix} t_{x}e^{i\Delta\phi^{\left( x \right)}} & r_{x}e^{-i\Delta\phi^{\left( x \right)}} \\ r_{x}e^{i\Delta\phi^{\left( x \right)}} & t_{x}e^{-i\Delta\phi^{\left( x \right)}} \end{matrix} \right)\left( \begin{matrix} t_{x}^{'} & r_{x}^{'} \\ r_{x}^{'} & t_{x}^{'} \end{matrix} \right)\boldsymbol{=}\left( \begin{matrix} Q_{11}^{eff} & Q_{12}^{eff} \\ Q_{21}^{eff} & Q_{22}^{eff} \end{matrix} \right)$ | (S31) |
| --- | --- | --- |

and we have

|  | $Q_{11}^{eff}=\frac{1}{4}\left( t_{x}t_{x}^{'}e^{i\Delta\phi^{\left( x \right)}}+r_{x}r_{x}^{'}e^{-i\Delta\phi^{\left( x \right)}} \right)=\frac{1}{4}\left[ \left( 2+\alpha_{0x} \right)\left( 1+\Delta\phi^{\left( x \right)} \right)+\left( 2-\alpha_{0x} \right)\left( 1-\Delta\phi^{\left( x \right)} \right) \right]=1+\frac{1}{2}\alpha_{0x}\Delta\phi^{\left( x \right)}$ | (S32) |
| --- | --- | --- |

Finally, by letting $Q_{11}=Q_{11}^{eff}$, we conclude that

|  | $\alpha_{01}\Delta\phi^{(1)}+\alpha_{02}\Delta\phi^{(2)}=\alpha_{0X}\Delta\phi^{(x)}$ | (S33) |
| --- | --- | --- |

which is in agreement with Eq. (S14).

Notice that if Eq. (S33) is satisfied, then the other three matrix elements of $Q$ and $Q^{eff}$ are also equal to each other. The derivations are similar and are therefore not provided here.

**3. Transmission and reflection coefficients (*S* parameters)**

An important difference between the GTTMM formalism we introduce here and that which we previously proposed [6] is the fact that we choose to use the electric displacement ($D$) rather than the electric field ($E$) to represent EM waves. Therefore, the matrix formalism, especially the calculation of the *S* parameters, needs to be modified. To accomplish this, let us follow the discussions in the Supplemental Document of [6], and derive the transmission and reflection coefficients in terms of the $D$ field, which we call the $D$-based *S* parameters.

An $N$-layer anisotropic material is ‘temporally embedded’ inside an isotropic homogeneous media (*e.g*., air), where $D_{s}^{i},D_{p}^{i},D_{s}^{r},D_{p}^{r}$ and $D_{s}^{t},D_{p}^{t}$ denote the complex amplitudes of the *s* and *p* modes of the incident, reflected, and transmitted waves, respectively (note that the temporal systems introduced in Section 4A, 4B, and 4C in the main text correspond to the case of $N=4, 1, 1$ respectively). The $D$ fields inside the last ($N+2$ th) temporal layer (determined by the parameters $D_{s}^{t},D_{p}^{t}$ $D_{s}^{r},$and $D_{p}^{r}$) and those inside the zeroth temporal layer ($D_{s}^{i}$ and $D_{p}^{i}$) are related by a transfer matrix given by

|  | $\left( \begin{matrix} D_{s}^{t} \\ D_{s}^{r} \\ D_{p}^{t} \\ D_{p}^{r} \end{matrix} \right)=Q\left( \begin{matrix} D_{s}^{i} \\ 0 \\ D_{p}^{i} \\ 0 \end{matrix} \right)=\left( \begin{matrix} Q_{11} & Q_{12} & Q_{13} & Q_{14} \\ Q_{21} & Q_{22} & Q_{23} & Q_{24} \\ Q_{31} & Q_{32} & Q_{33} & Q_{34} \\ Q_{41} & Q_{42} & Q_{43} & Q_{44} \end{matrix} \right)\left( \begin{matrix} D_{s}^{i} \\ 0 \\ D_{p}^{i} \\ 0 \end{matrix} \right)$ | (S34) |
| --- | --- | --- |

This equation is identical to Eq. (S9) in [6] except that the $E$ fields are replaced by $D$ fields.

Accordingly, the $D$-based *S* parameters can be expressed as (suppose we consider that the incident wave is $p-$polarized):

|  | $t_{ps}^{D}=\frac{D_{s}^{t}}{D_{p}^{i}}=Q_{13}, t_{pp}^{D}=\frac{D_{p}^{t}}{D_{p}^{i}}=Q_{33}$  $r_{ps}^{D}=\frac{D_{s}^{r}}{D_{p}^{i}}=Q_{23}, r_{pp}^{D}=\frac{D_{p}^{r}}{D_{p}^{i}}=Q_{43}$ | (S35) |
| --- | --- | --- |

where $D_{s}^{i}=0$ is assumed. Here we use the superscript $'D'$ to denote that our notation is different from that used in the conventional *E*-based approach (Eq. (S10) in  [6]).

It is obvious that these two sets of notations are related in the following way:

|  | $t_{ps}=\frac{E_{s}^{t}}{E_{p}^{i}}=\frac{D_{s}^{t}/\varepsilon^{(N+2)}}{D_{p}^{i}/\varepsilon^{(1)}}{=t}_{ps}^{D}\cdot\frac{\varepsilon^{\left( 1 \right)}}{\varepsilon^{(N+2)}}$ | (S36) |
| --- | --- | --- |

|  | $t_{pp}=\frac{E_{p}^{t}}{E_{p}^{i}}=\frac{D_{p}^{t}/\varepsilon^{(N+2)}}{D_{p}^{i}/\varepsilon^{(1)}}{=t}_{pp}^{D}\cdot\frac{\varepsilon^{\left( 1 \right)}}{\varepsilon^{(N+2)}}$ | (S37) |
| --- | --- | --- |

and the expressions for $r_{ps}$ and $r_{pp}$ can be calculated in the same fashion.

For the first three temporal systems considered in the main text (Section 4A, 4B and 4C), we can obtain the desired *S* parameters by using Eq. (S36) and Eq. (S37).

**4. Derivation of conditions for an anisotropic anti-reflection time coating (AATC)**

In Section 4C of the main text, where we introduce anisotropic anti-reflection temporal coatings, we present the conditions for reflection cancellation. Here, we provide a more detailed derivation for these conditions.

We begin with the expression for the $Q$ matrix of this system:

|  | $Q=\mathcal{D}_{1}^{-1}\mathcal{D}_{2}P_{2}\mathcal{D}_{2}^{-1}\mathcal{D}_{3}$ | (S38) |
| --- | --- | --- |

where the definition of the $\mathcal{D}$ and $P$ matrices have been given in Eq. (12.1) and Eq. (9), respectively, in the main text.

After some mathematical manipulations, we can express the *S* parameters (the elements of the $Q$ matrix) as functions of the parameters of each temporal layer (*i.e*., $\varepsilon^{\left( i \right)},\Delta t^{(i)}$, $i=1,2,3$), and the angles $\theta$ and $\phi$:

|  | $r_{ss}^{D}=Q_{21}, r_{sp}^{D}=Q_{41},r_{pp}^{D}=Q_{43}, r_{ps}^{D}=Q_{23}$ | (S39) |
| --- | --- | --- |

|  | $r_{ss}=r_{ss}^{D}\frac{\varepsilon^{\left( 1 \right)}}{\varepsilon^{\left( 3 \right)}}=Q_{21}\frac{\varepsilon^{\left( 1 \right)}}{\varepsilon^{\left( 3 \right)}}=\left( 1-\frac{Z^{'(1)}}{Z^{'(3)}} \right)\left( s_{2}^{2}\cos\left( \omega_{1}T \right)+c_{2}^{2}\cos\left( \omega_{3}T \right) \right)\frac{\varepsilon^{\left( 1 \right)}}{\varepsilon^{\left( 3 \right)}}$  $+i\left[ s_{2}^{2}\left( \frac{Z^{'(1)}}{Z_{y}^{'(2)}}-\frac{Z_{y}^{'(2)}}{Z^{'(3)}} \right)\sin\left( \omega_{1}T \right)+c_{2}^{2}\left( \frac{Z^{'(1)}}{Z_{x}^{'(2)}}-\frac{Z_{x}^{'(2)}}{Z^{'(3)}} \right)\sin\left( \omega_{3}T \right) \right]\frac{\varepsilon^{\left( 1 \right)}}{\varepsilon^{\left( 3 \right)}}=0$ | (S40) |
| --- | --- | --- |

|  | $r_{pp}=r_{pp}^{D}\frac{\varepsilon^{\left( 1 \right)}}{\varepsilon^{\left( 3 \right)}}=Q_{43}\frac{\varepsilon^{\left( 1 \right)}}{\varepsilon^{\left( 3 \right)}}=\left( 1-\frac{Z^{'(1)}}{Z^{'(3)}} \right)\left( s_{2}^{2}\cos\left( \omega_{1}T \right)+c_{2}^{2}\cos\left( \omega_{3}T \right) \right)\frac{\varepsilon^{\left( 1 \right)}}{\varepsilon^{\left( 3 \right)}}$  $+i\left[ c_{2}^{2}\left( \frac{Z^{'(1)}}{Z_{y}^{'(2)}}-\frac{Z_{y}^{'(2)}}{Z^{'(3)}} \right)\sin\left( \omega_{1}T \right)+s_{2}^{2}\left( \frac{Z^{'(1)}}{Z_{x}^{'(2)}}-\frac{Z_{x}^{'(2)}}{Z^{'(3)}} \right)\sin\left( \omega_{3}T \right) \right]\frac{\varepsilon^{\left( 1 \right)}}{\varepsilon^{\left( 3 \right)}}=0$ | (S41) |
| --- | --- | --- |

|  | $r_{sp}=r_{sp}^{D}\frac{\varepsilon^{\left( 1 \right)}}{\varepsilon^{\left( 3 \right)}}=Q_{41}\frac{\varepsilon^{\left( 1 \right)}}{\varepsilon^{\left( 3 \right)}}=\frac{c_{2}s_{2}}{4}\{\left( 1-\frac{Z_{1}}{Z_{3}} \right)\left[ \cos\left( \omega_{1}T \right)-\cos\left( \omega_{3}T \right) \right]\frac{\varepsilon^{\left( 1 \right)}}{\varepsilon^{\left( 3 \right)}}$  $+i\left[ \left( \frac{Z^{'(1)}}{Z_{y}^{'(2)}}-\frac{Z_{y}^{'(2)}}{Z^{'(3)}} \right)\sin\left( \omega_{1}T \right)-\left( \frac{Z^{'(1)}}{Z_{x}^{'(2)}}-\frac{Z_{x}^{'(2)}}{Z^{'(3)}} \right)\sin\left( \omega_{3}T \right) \right]\frac{\varepsilon^{\left( 1 \right)}}{\varepsilon^{\left( 3 \right)}}\}=0$ | (S42) |
| --- | --- | --- |

Here we do not show the expression for $r_{ps}$, which can be easily proven to be equal to $r_{ps}$. Also, $\omega_{1}$ and $\omega_{3}$ have been defined in Eq. (11.1) and (11.2), respectively, in the main text (the superscript ^(2)^is omitted here):

|  | $\omega_{1}^{\left( i \right)}=ck\sqrt{\frac{c_{1}^{2}\mu_{Z}^{\left( i \right)}+s_{1}^{2}\mu_{X}^{\left( i \right)}}{\varepsilon_{Y}^{(i)}\mu_{X}^{(i)}\mu_{Z}^{(i)}}}=\frac{ck}{n_{y}^{'(i)}}=\frac{2\pi f}{n_{y}^{'(i)}}$  $\omega_{3}^{\left( i \right)}=ck\sqrt{\frac{c_{1}^{2}\varepsilon_{Z}^{\left( i \right)}+s_{1}^{2}\varepsilon_{X}^{\left( i \right)}}{\varepsilon_{X}^{(i)}\varepsilon_{Z}^{(i)}\mu_{Y}^{(i)}}}=\frac{ck}{n_{x}^{'(i)}}=\frac{2\pi f}{n_{x}^{'(i)}}$ | (S43) |
| --- | --- | --- |

where $f$ is the frequency of the incident wave.

In order that the above four equations be equal to zero simultaneously, for any given incident wave (*i.e*., $c_{1}, c_{2}, s_{1}, \mathrm{and} s_{2}$ can vary), it is obvious that the following conditions must be satisfied:

|  | $\frac{Z^{'(1)}}{Z_{y}^{'(2)}}-\frac{Z_{y}^{'(2)}}{Z^{'(3)}}=\frac{Z^{'(1)}}{Z_{x}^{'(2)}}-\frac{Z_{x}^{'(2)}}{Z^{'(3)}}=0$  $\cos\left( \omega_{1}^{(2)}T \right)=\cos\left( \omega_{3}^{(2)}T \right)=0$ | (S44) |
| --- | --- | --- |

Assuming the material is non-magnetic, and using Eq. (12.3) in the main text, Eq. (S44) can be rewritten as:

|  | $\frac{c_{1}^{2}}{\varepsilon_{X}^{(2)}}+\frac{s_{1}^{2}}{\varepsilon_{Z}^{(2)}}=\frac{1}{\varepsilon_{Y}^{(2)}}=\frac{1}{\sqrt{\varepsilon^{(1)}\varepsilon^{(3)}}}$  $\omega_{1}^{(2)}T=\omega_{3}^{(2)}T=\pi/2$ | (S45) |
| --- | --- | --- |

which agrees with Eq. (19) in the main text.

It follows that $\omega_{1}^{(2)}$ and $\omega_{3}^{(2)}$ are proportional to $f$, so Eq. (S45) only holds for a certain frequency $f_{0}$. When $f\neq f_{0}$, we have $\cos\left( \omega_{1}T \right)\neq0, \cos\left( \omega_{3}T \right)\neq0$, therefore, $Q_{21}\neq0$ and $Q_{43}\neq0$. However, the condition $\cos\left( \omega_{1}T \right)-\cos\left( \omega_{3}T \right)=0$ is still valid, so $Q_{41}=Q_{23}=0$. This provides a brief explanation of why $r_{ps}=r_{sp}=0$ over the entire frequency range rather than just at $f=f_{0}$.

**5. The calculation of** $\boldsymbol{\Delta}\boldsymbol{\theta}_{\boldsymbol{s}}$ **in Section 4D**

In this section, we derive the expression for $\Delta\theta_{s}$ (Eq. (22)), which appears in Section 4D of the main text where we discuss the redirection of Poynting vectors.

When the medium is anisotropic ($t>t^{(1)}$), the electric and magnetic fields of the transmitted wave can be expressed as

|  | $\boldsymbol{E}=\left( E_{x}, E_{y}, E_{z} \right) \boldsymbol{H}=\left( H_{x}, H_{y}, H_{z} \right)$  $\boldsymbol{D}=\left( D_{x}, D_{y},0 \right)=D_{0}\cdot\left( c_{2},s_{2},0 \right)$  $\boldsymbol{B}=\left( B_{x}, B_{y},0 \right)=B_{0}\cdot\left( -s_{2},c_{2},0 \right)$ | (S46) |
| --- | --- | --- |

where $D_{0}$ and $B_{0}$ are the magnitudes of the $\boldsymbol{D}$ and $\boldsymbol{B}$ fields. $\boldsymbol{D},$ $\boldsymbol{B},$ $\boldsymbol{E}$ and $\boldsymbol{H}$ are related by

|  | $\boldsymbol{E}=\delta\boldsymbol{D H=}\eta\boldsymbol{B}$ | (S47) |
| --- | --- | --- |

Notice that in the main text, $t>t^{(1)}$ corresponds to the 2^nd^ temporal layer, so the fields and the Poynting vector are labelled with the superscript ^(2)^. In this document, however, we drop all the superscripts in order to simplify the notation.

First, we recognize that the three components of the Poynting vector can be expressed as

|  | $S_{x}=E_{y}H_{z}-E_{z}H_{y}$ $=\delta_{yy}D_{y}\eta_{zx}B_{x}-\delta_{zx}D_{x}\eta_{yy}B_{y}$ $=\frac{1}{\varepsilon_{Y}}\left[ -\frac{c_{1} s_{1}}{\mu_{X}\mu_{Z}}\left( \mu_{Z}-\mu_{X} \right) \right]\cdot\left( -s_{2}^{2} \right)D_{0}B_{0}-\frac{1}{\mu_{Y}}\left[ -\frac{c_{1} s_{1}}{\mu_{X}\mu_{Z}}\left( \mu_{Z}-\mu_{X} \right) \right]\cdot c_{2}^{2} D_{0}B_{0}$ $=c_{1}s_{1}\left[ \frac{s_{2}^{2}}{\varepsilon_{Y}}\left( \frac{1}{\mu_{X}}-\frac{1}{\mu_{Z}} \right)+\frac{c_{2}^{2}}{\mu_{Y}}\left( \frac{1}{\varepsilon_{X}}-\frac{1}{\varepsilon_{Z}} \right) \right]D_{0}B_{0}$ | (S48) | |  |
| --- | --- | --- | --- | --- |
|  | $S_{y}=E_{z}H_{x}-E_{x}H_{z}$ $=\delta_{zx}D_{x}\eta_{xx}B_{x}-\delta_{xx}D_{x}\eta_{zx}B_{x}$ $=\left( -\frac{c_{1}s_{1}}{\varepsilon_{X}\varepsilon_{Z}}\left( \varepsilon_{Z}-\varepsilon_{X} \right) \right)\cdot\left( \frac{c_{1}^{2}\mu_{z}+s_{1}^{2}\mu_{x}}{\mu_{X}\mu_{Z}} \right)\cdot\left( -c_{2}s_{2}D_{0}B_{0} \right)-\frac{c_{1}^{2} \varepsilon_{Z}+s_{1}^{2}\varepsilon_{X}}{\varepsilon_{Z}\varepsilon_{X}}\left( -\frac{c_{1}s_{1}}{\mu_{X}\mu_{Z}}\left( \mu_{Z}-\mu_{X} \right) \right)\cdot c_{2}s_{2}D_{0}B_{0}$ $=c_{1}s_{1}c_{2}s_{2}\left[ \left( \frac{1}{\varepsilon_{X}}-\frac{1}{\varepsilon_{Z}} \right)\left( \frac{c_{1}^{2}}{\mu_{X}}+\frac{s_{1}^{2}}{\mu_{Z}} \right)+\left( \frac{c_{1}^{2}}{\varepsilon_{X}}+\frac{s_{1}^{2}}{\varepsilon_{Z}} \right)\left( \frac{1}{\mu_{X}}-\frac{1}{\mu_{Z}} \right) \right]D_{0}B_{0}$ | | (S49) | |

|  | $S_{z}=E_{x}H_{y}-E_{y}H_{x}$ $=\delta_{xx}D_{x}\eta_{yy}B_{y}-\delta_{yy}D_{y}\eta_{xx}B_{x}$ $\left[ \frac{1}{\varepsilon_{X}\varepsilon_{Z}\mu_{Y}}\left( c_{1}^{2}\varepsilon_{Z}+s_{1}^{2}\varepsilon_{X} \right)c_{2}^{2}+\frac{1}{\mu_{X}\mu_{Z}\varepsilon_{Y}}\left( c_{1}^{2}\mu_{Z}+s_{1}^{2}\mu_{X} \right)s_{2}^{2} \right]D_{0}B_{0}$ | (S50) |
| --- | --- | --- |

We note that these general expressions for $S_{x}, S_{y}$ and $S_{z}$ are quite complicated. Next, we assume that the material is non-magnetic (*i.e*., $\mu_{X}=\mu_{Y}=\mu_{Z}=1$). With this simplification, $\boldsymbol{S}$ can be written as

|  | $S_{x}=c_{1}s_{1}c_{2}^{2}\left( \frac{1}{\varepsilon_{X}}-\frac{1}{\varepsilon_{Z}} \right)D_{0}B_{0}$  $S_{y}=c_{1}s_{1}c_{2}s_{2}\left( \frac{1}{\varepsilon_{X}}-\frac{1}{\varepsilon_{Z}} \right)D_{0}B_{0}$  $S_{z}=\left[ \frac{1}{\varepsilon_{x}\varepsilon_{z}}\left( c_{1}^{2}\varepsilon_{z}+s_{1}^{2}\varepsilon_{x} \right)c_{2}^{2}+\frac{1}{\varepsilon_{y}}s_{2}^{2} \right]D_{0}B_{0}$ | (S51) |
| --- | --- | --- |

Therefore,

|  | $\Delta\theta_{S}=\tan^{-1} \left( \frac{\sqrt{S_{x}^{2}+S_{y}^{2}}}{S_{z}} \right)=\frac{c_{1}c_{2}s_{1}\left( \frac{1}{\varepsilon_{X}}-\frac{1}{\varepsilon_{Z}} \right)}{\frac{1}{\varepsilon_{x}\varepsilon_{z}}\left( c_{1}^{2}\varepsilon_{z}+s_{1}^{2}\varepsilon_{x} \right)c_{2}^{2}+\frac{1}{\varepsilon_{y}}s_{2}^{2}}$ | (S52) |
| --- | --- | --- |

which agrees with Eq. (22) in the main text.

**References**

[1] T. C. Choy, *Effective Medium Theory: Principles and Applications* (2016).

[2] E. Lustig, Y. Sharabi, and M. Segev, *Topological Aspects of Photonic Time Crystals*, Optica **5**, 1390 (2018).

[3] V. Pacheco-Peña and N. Engheta, *Effective Medium Concept in Temporal Metamaterials*, Nanophotonics **9**, 379 (2020).

[4] J. D. Joannopoulos, P. R. Villeneuve, and S. Fan, *Photonic Crystals: Putting a New Twist on Light*, Nature **386**, 143 (1997).

[5] J. Li, L. Zhou, C. T. Chan, and P. Sheng, *Photonic Band Gap from a Stack of Positive and Negative Index Materials*, Phys. Rev. Lett. **90**, 083901 (2003).

[6] J. Xu, W. Mai, and D. H. Werner, *Complete Polarization Conversion Using Anisotropic Temporal Slabs*, Opt. Lett. **46**, 1373 (2021).
